# Identifying the Thermodynamic Driving Force of Metal Extraction by Hydrophobic Eutectic Solvents

**DOI:** 10.1002/cssc.70794

**Published:** 2026-06-07

**Authors:** Inês C. M. Vaz, Maísa Saldanha Pinheiro, Felipe Olea, Luigi Cirillo, Giorgia Mannucci, Matteo Busato, Paola D’Angelo, Rui Santos, Margarida Bastos, Luís M. N. B. F. Santos, João A. P. Coutinho, Nicolas Schaeffer

**Affiliations:** ^1^ CICECO – Aveiro Institute of Materials Department of Chemistry University of Aveiro Aveiro Portugal; ^2^ Dipartimento di Chimica Università degli Studi di Roma La Sapienza Rome Italy; ^3^ Analytik Jena GmbH at Centro de Investigação em Química (CIQUP) Faculdade de Ciências da Universidade do Porto Porto Portugal; ^4^ Centro de Investigação em Química (CIQUP) Institute of Molecular Sciences (IMS) Departamento de Química e Bioquímica Faculdade de Ciências da Universidade do Porto Porto Portugal

**Keywords:** calorimetry, hydrometallurgy, lanthanides, solvent extraction, thermodynamics

## Abstract

The biphasic transfer of Eu(NO_3_)_3_ by trioctylphosphine oxide (TOPO) diluted in a molecular diluent or as a component of a hydrophobic eutectic solvent (HES) was studied by two‐phase isothermal titration calorimetry and complemented by XAS, FTIR, and NMR spectroscopy. In HES, the solvent intermolecular interactions introduce an enthalpic penalty, which is overcompensated by a reduction of the entropic cost of Eu(III) phase transfer, resulting in enhanced metal partitioning.

## Graphical abstract

Solvent extraction (SX) in hydrophobic eutectic solvent (HES) appears as an extension rather than a “revolution” of SX in common organic diluents for a given neutral extractant. A detailed thermodynamic and spectroscopic study shows that SX in HES imposes an enthalpic penalty but lowers the entropy cost of Eu^3+^ transfer, leading to enhanced metal partitioning.

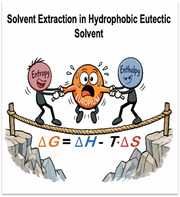

## 1 Introduction

New designs and the improvement of existing separation processes are required to address the dual problem of rising primary metal demand and electronic waste volumes for recycling [1, 2]. Among the various options, solvent extraction (SX) stands out due to its versatility and industrial maturity [3]. SX relies on the selective biphasic transfer of a target metal ion from an aqueous to organic phase, facilitated by its chelation with an extractant molecule to overcome the energetic barrier to ion dehydration and stabilize the resulting complex in the apolar diluent. Recently, nonionic hydrophobic eutectic solvents (HESs) were proposed as substitutes for the conventional organic phase in SX [4]. Importantly, unlike common diluents, HESs are liquid mixtures formed by combining hydrogen bond donors (HBDs) with acceptors (HBAs) allowing the liquefaction of one of them through solid–liquid equilibrium (SLE). By incorporating an extractant molecule as part of a binary HES mixture, perceived advantages of HES for SX include (i) the increased extractant solubility via liquefaction, (ii) the absence of third‐phase formation, and (iii) the elimination of petroleum‐derived diluents and phase modifiers [5]. Promising results on the SX application of nonionic HES containing varied extractant types, including those based on carboxylic acids [6], trialkylphosphine oxides [7], heterocyclic organic compounds [8], β‐diketones [9], or diglycolamides [10], were reported. However, it is not yet clear if the underlying extraction mechanism differs for a given extractant when used in a HES or in a molecular diluent. This is particularly relevant for mixtures presenting strong thermodynamic nonideality, as it is expected that intermolecular interactions in HES play a significant role in SX in comparison with a diluted extractant solution [11].

To develop new extraction systems and move beyond trial‐and‐error application of HES toward their rational design, a fundamental understanding of variations in distribution coefficients (*D*_M_) and selectivity relative to conventional SX is required. While many experimental or theoretical approaches are available, few can bridge the range of lengths and timescales relevant to describe the entire SX phenomena [12]. Thermodynamics can provide insight into extraction mechanisms, with the overall Gibbs energy of extraction (Δ*G*_ex_) reflecting the energy changes of various steps occurring during SX including ion dehydration, ion–ligand complexation, complex interfacial transfer, and complex solvation in the organic phase via solvent restructuration [13]. Even when the enthalpy of extraction (Δ*H*_e__x_) is not the primary driving force, its accurate determination is critical for correctly determining the entropic contribution (Δ*S*_ex_) to ion partitioning. Due to its experimental simplicity and accessibility, the van’t Hoff relation is commonly employed, as it only requires equilibrium data obtained at different temperatures (ln *K*_ex_ vs. 1/*T*). While reasonable for extractions based on a simple equilibrium, the van’t Hoff approach is often unable to capture nonlinear effects due to complex equilibria, as commonly observed in mixed extractant systems [14]. Furthermore, given the temperature dependent nonideality of these mixtures, the validity of the van’t Hoff relation for HES cannot be assumed and remains to be demonstrated [11].

A more rigorous determination of Δ*H*_ex_ can be done through the calorimetric determination of the heat of extraction. While this approach was reported in the 1970s by Marcus and Kolarik [15] and reproduced by others since [16, 17, 18, 19], its application remains limited and to the best of our knowledge was not yet applied to the study of SX in HES. In this work, isothermal titration calorimetry (ITC) is used for the first time to compare the thermodynamic signatures associated with the biphasic transfer of trisnitrato complexes of europium (Eu(NO_3_)_3_) by a solvating ligand (tri‐n‐octylphosphine oxide, TOPO) either incorporated in a HES with decanoic acid (C_9_H_19_COOH) as HBD for *x*_TOPO_ = 0.5, or diluted in a molecular diluent (200 mmol dm^−3^ TOPO in toluene). The Eu(NO_3_)_3_/TOPO combination was selected because it was previously studied by ITC, allowing for method validation [16], while the HES system is well‐characterized, thereby facilitating the interpretation of results [20]. The aim is to understand the thermodynamic driving forces of the extraction and what they reveal about the underlying mechanisms of SX in HES.

## 2 Results and Discussion

**FIGURE 1:**
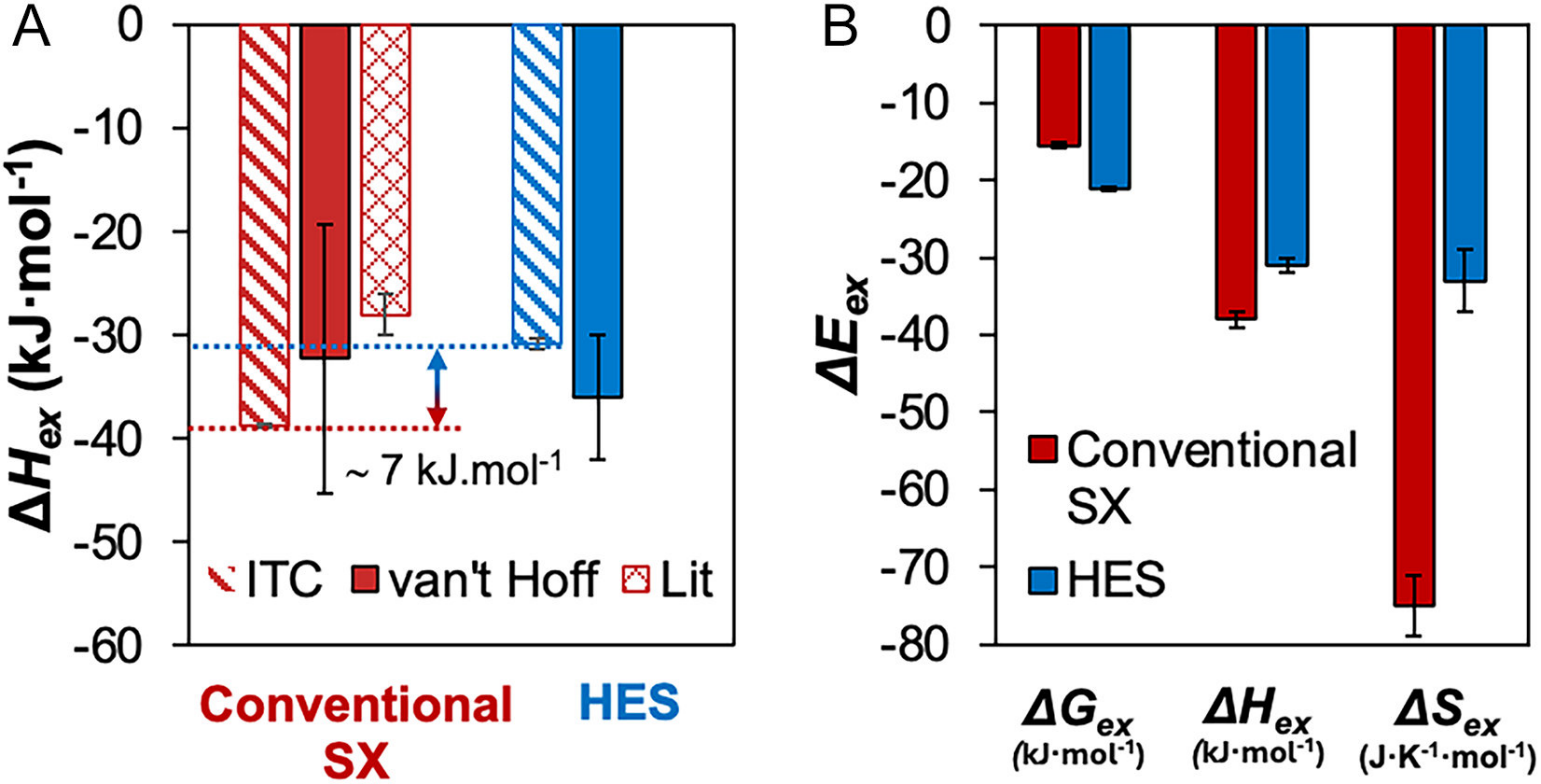
(A) Δ*H*_ex_ of Eu(NO_3_)_3_ in the conventional SX (200 mmol·dm^−3^ TOPO in toluene) and HES system (TOPO + decanoic acid, *x*_TOPO_ = 0.5) as measured by ITC and the van’t Hoff method, and compared with the ITC result of Grimes et al. [16]. (B) Comparison of the contributions to Eu^3+^ SX in the conventional and HES phases based on the Δ*H*_ex_ values from ITC and Eu^3+^ partition at 298 K.

As Δ*H*_ex_ measured by ITC comprises composite heats arising from multiple individual processes, it is important to differentiate Eu^3+^ extraction from the coextraction of other solutes (H_2_O and HNO_3_). More details regarding the methodology and experimental procedure are available in the ESI (Tables S2 and S3, Figures S1–S3). The obtained Δ*H*_ex_ for Eu(NO_3_)_3_ in the conventional SX system and in the HES phase are presented in Figure 1A. The results are evaluated against experimental data obtained via equilibrium measurements and the van’t Hoff relation (hereafter referred to as “van’t Hoff method,” details in Tables S4 and S5 and Figures S4 and S5) as well as against those reported in the literature for the extraction of Eu^3+^ by TOPO from nitrate media, all summarized in Table 1. The biphasic Δ*H*_ex_ from ITC in both the conventional diluent and HES both indicate that the extraction of Eu(NO_3_)_3_ is an exothermic process, although less so in the HES than in conventional SX by approximately 7 kJ·mol^−1^. The less exothermic Δ*H*_ex_ obtained for HES can be rationalized by the presence of secondary temperature‐dependent interactions in the mixture, such as hydrogen bonding between TOPO and decanoic acid. This additional endothermic contribution may arise from the partial disruption of the HES intermolecular hydrogen bonding due to competition between Eu^3+^ and decanoic acid for interaction with TOPO, as supported by the spectroscopic results below.

**TABLE 1:** Extraction heats for Eu^3+^ in nitrate media for the conventional SX and HES phases as measured by the van’t Hoff method and biphasic ITC.

**Δ*H*_ex_, kJ·mol** ^**−1**^	Conventional SX	HES
ITC (this work)^a^	−38.4 ± 0.6	−31 ± 1
ITC (literature)	−29.4 ± 1.5 [16]	—
van’t Hoff (this work)^b^	−32 ± 13	−36 ± 6
van’t Hoff (literature)	−28.0 to −61.7 [16, 21, 22, 23]	−8.6 [20]

a Enthalpy is presented ±$2\times \sigma /\sqrt{n}$, where σ is the standard deviation of the values obtained from the independent measurements and *n* is the number of independent ITC measurements.

b Enthalpy is presented ±$2\times \sigma /\sqrt{n}$, where σ is the standard deviation of the slope of ln *K* vs. 1000/*T* and *n* is the number of temperatures.

In both the conventional SX and HES systems, Δ*H*_ex_ values obtained by ITC and van’t Hoff method under the same system composition are in good agreement, further validating the measurements. A wider comparison with the literature (Table 1) indicates a large variability, which could be due to the different aqueous phase compositions, as well as the organic phase extractant concentration and purity [19, 21]. For example, the Δ*H*_ex_ for lanthanide nitrate extraction varied from −50 to −35 kJ·mol^−1^ as the TOPO concentration decreased from 0.07 to 0.03 mol·dm^−3^ [21]. Despite the anticipated limitations of the van’t Hoff analysis for nonideal extractant systems, a similar value within experimental error to ITC is obtained. This cannot be expected to apply for all HES systems as the two methods probe different thermodynamic observables: van’t Hoff reflects only the temperature dependence of the extraction equilibrium, whereas ITC directly measures the total heat exchanged during extraction, including interaction and reorganization processes.

Comparison with reported ITC measurements of the conventional SX system for the same TOPO concentration and aqueous phase composition [16] reveals a small difference. This difference is assigned to the explicit quantification of the degree of hydration of the initial Eu(NO_3_)_3_·nH_2_O salt in this study. If the Eu(NO_3_)_3_ salt is considered to be anhydrous, the obtained Δ*H*_ex_ decreases to –28 ± 2 kJ·mol^−1^, consistent with the values reported by Grimes et al. [16] within the experimental uncertainty (see results in the Supporting Information). Importantly, previous results showed comparable Δ*H*_ex_ across the lanthanide series [16, 21], suggesting that the associated discussion can be extrapolated to the extraction of other Ln^3+^ in TOPO‐based HES from nitrate media.

From the obtained Δ*H*_ex_ by ITC and measured distribution coefficients, the thermodynamic parameters for the extraction of Eu(NO_3_)_3_ in conventional SX and HES are presented in Figure 1B (see the “Thermodynamic Analysis” section of the Supporting Information). It should be noted that here the standard state of Δ*G*_ex_ is defined for the solvent containing all the species but the Eu(NO_3_)_3_ salt and represents the Gibbs energy per mole of transferred Eu^3+^. Therefore, it is not the raw difference of a given sample versus a reference state, as classically noted by ΔG^0^. Extraction in both systems is enthalpically driven, in which the Eu^3+^−TOPO interaction, favored by the strong electronegativity of the P=O bond, overcomes the energetic barrier to Eu^3+^ dehydration and solvation. The magnitude of the Δ*G*_e__x_, which is similar in both solvent phases, is quenched to varying extents by an unfavorable entropic contribution term. For extraction in the conventional SX, the entropic penalty was assigned to the higher degree of ordering required to accommodate Eu^3+^ complexation with three nitrate anions and solvation by three TOPO molecules in the organic phase (complex identified from the literature) [16, 21, 22, 23].

However, this explanation alone does not account for the nearly twofold reduction of the Δ*S*_ex_ term in the HES relative to the conventional SX, even when considering that nitric acid coextraction is only moderately higher in the former (see solute coextraction in Tables S6 and S7). Additional structural effects must be considered. In particular, the significantly higher TOPO concentration and the presence of hydrogen‐bond interactions between TOPO and decanoic acid result in a more preorganized extractant phase. Consequently, the formation of the Eu–TOPO complex requires a smaller loss of configurational entropy in HES than in conventional SX, where TOPO molecules must reorganize from a more dilute and disordered state. The higher degree of preorganization in the HES thus contributes to a reduced entropic penalty upon extraction. Moreover, the reduction in Δ*S*_ex_ observed is consistent in magnitude with that expected solely from the higher TOPO concentration in the HES system. Assuming that the entropic contribution associated with complex formation scales with the configurational term –*n*·*R*·ln[TOPO], where *n* corresponds to the number of TOPO molecules coordinated to Eu^3+^, the resulting ΔΔ*S*_ex_ between HES and conventional SX (ΔΔ*S*_ex _= Δ*S*_ex_(HES) – Δ*S*_ex_(SX) = 51 J·mol^−1^·K^−1^) is of the same order of magnitude as that experimentally determined (ΔΔ*S*_ex_ = 43 J·mol^−1^·K^−1^). In this calculation, the total TOPO concentration in the HES phase was considered. However, this likely overestimates the available extractant, as a portion of TOPO is engaged in hydrogen‐bond interactions within the HES. The hindered availability of the extractant would explain the lower experimental ΔΔ*S*_ex_. Nevertheless, while the higher TOPO concentration provides a quantitative rationale for the reduced entropic penalty, relying on this factor alone would be an oversimplification.

**FIGURE 2:**
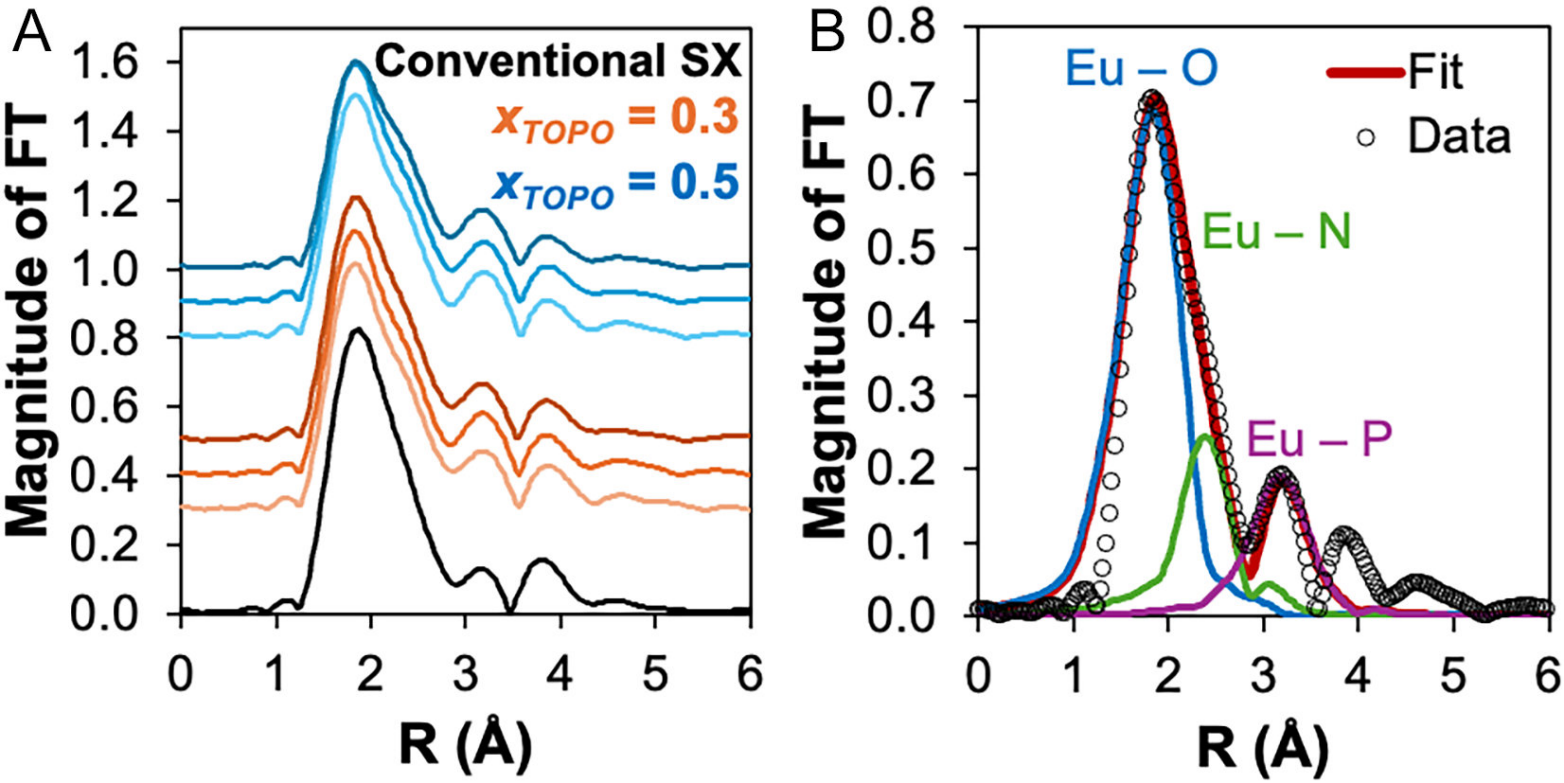
(A) Nonphase shift corrected Fourier transform of the EXAFS experimental data of Eu^3+^ in the conventional SX system, and in the HES phase for *x*_TOPO_ = 0.3 or 0.5 after contact with three initial aqueous phase Eu(NO_3_)_3_ concentrations (0.025, 0.071, and 0.152 mol·dm^−3^; color code from light to dark reflects the concentration increase). (B) Fitted FT theoretical signals (red line) for the TOPO + decanoic acid HES (*x*_TOPO_ = 0.5), ([Eu^3+^]_HES_ = 0.05 mol·dm^−3^) showing the Eu–X (X = O, N, P) paths.

Beyond the entropic considerations discussed above, the less exothermic Δ*H*_ex_ and smaller entropic penalty observed for the HES systems may also be hypothesized to arise from (i) speciation, which until now was assumed to be identical in both organic phases as the complex Eu(NO_3_)_3_(TOPO)_3_, and/or (ii) solvent effects arising from the nature of HES interactions and its structuration. Steric hindrance in the HES relative to the conventional SX could promote the formation of the Eu(NO_3_)_3_(TOPO)_2_ species, thereby lowering Δ*H*_ex_ due to the reduction of Eu^3+^−TOPO coordination while concomitantly reducing the entropic term as one less TOPO molecule is involved in the extraction. To validate this hypothesis, X‐ray absorption spectroscopy (XAS) data were collected at the Eu^3+^ L_3_‐edge for the conventional SX and HES phases after extraction. More details on the experimental and analysis procedure are available in the “Europium speciation” section of the ESI (Tables S8–S10, Figures S7 and S8). To ensure that the HES results are representative and not condition specific, three different Eu^3+^ concentrations (from 0.05 to 0.22 mol·dm^−3^, see Table S8) were evaluated for two different *x*_TOPO_ compositions of 0.5 and 0.3. The EXAFS experimental spectra shown in Figure S7, together with the corresponding Fourier transforms (FT) calculated over the 2.7–10.7 Å^−1^ *k*‐range for all the investigated systems (Figure 2A), are remarkably similar. These results indicate that the Eu^3+^ ion maintains the same short‐range local structural arrangement across all samples. As in the conventional SX, the inner‐sphere coordination environment around Eu^3+^ in the HES is dominated by TOPO–cation interaction with no indication of secondary contributions from decanoic acid even when present in excess (*x*_TOPO_ = 0.3). Under the aqueous phase conditions used during SX (pH ∼ 1), no deprotonation of decanoic acid is expected (pKa = 4.9), consistent with the absence of observed Eu^3+^–carboxylate interactions. Furthermore, the Eu^3+^ local environment appears to be relatively insensitive to the HES phase metal loading, simplifying the analysis.

The EXAFS data were analyzed including the Eu–O, Eu–N, and Eu–P two‐body contributions up to a distance of 3.8 Å, as determined from the crystal structure of tris(triethylphosphine oxide) europium tris(nitrato) (CCDC 866501) [24]. The FT best‐fit results are shown in Figure 2B, and the agreement between the experimental and theoretical curve is satisfactory. As no differences were observed among HES samples, only the best‐fit structural parameters for the conventional SX phase and *x*_TOPO_ = 0.5 for [Eu^3+^]_org_ = 0.05 mol·dm^−3^ are presented in Table 2. The obtained bond distances in the conventional SX systems agree with those of the crystal structure, the latter presenting an average Eu–O length of 2.442 ± 0.117 Å, Eu–N length of 2.951 ± 0.001 Å, and Eu–P length of 3.757 ± 0.016 Å. The average Eu–O bond length in the crystal structure is the contribution of a shorter Eu–TOPO interaction at 2.290 Å and longer Eu–NO_3_^−^ at 2.518 Å [24]. However, the introduction of two distinct Eu–O contributions did not improve the EXAFS fit.

**TABLE 2:** Structural and fitting parameters derived from the EXAFS analysis of Eu^3+^ in the conventional SX system and acid HES phase (*x*_TOPO_ = 0.5) for [Eu^3+^]_org_ = 0.05 mol dm^−3^. Associated uncertainty values are available in Tables S8 and S9.

Path	Conventional SX			HES		
***N***	*R*, Å	***σ*** ^**2**^ **,** **Å** ^**–2**^	***N***	*R*, Å	***σ*** ^**2**^ **,** **Å** ^**–2**^
Eu–O	8.3	2.38	0.009	9.1	2.37	0.013
Eu–N	3.0^a^	2.94	0.001	3.0^a^	2.93	0.002
Eu–P	2.3	3.76	0.006	3.2	3.78	0.006

*Note:* Coordination number *N*, average distance *R*, Debye–Waller factor *σ* ^2^.

a The coordination number of nitrate was fixed to 3.

Interestingly, no significant differences in the Eu–O bond lengths are observed between the conventional SX and HES phases (2.38–2.37 Å respectively), suggesting that the complex appears consistent across the systems despite the differing nature of the solvents. The same is true for the complex stoichiometry despite an increasing magnitude of the Eu–P path in the HES. The minimal error of about ± 1 associated with coordination number determination by EXAFS prevents further analysis, future work will address the XANES portion of the XAS spectra to confirm the complex geometry. As in previous time‐resolved luminescence spectroscopy analysis of Eu^3+^ in the same HES, no presence of inner‐sphere water molecules was found [20], and the most probable coordination mode of the nitrate anions is bidentate to satisfy the assumed 9‐coordinate complex.

**FIGURE 3:**
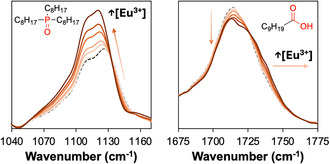
FTIR spectra of the HES phase for *x*_TOPO_ = 0.5, focusing on the P=O stretching band of TOPO (left) and C=O stretching band of decanoic acid (right) as a function of the [Eu^3+^]_HES_ concentration after extraction from 0 to 35 g·dm^−3^. All absorbances are standardized to the CH_2_ mode at 2922 cm^−1^. The black dashed line represents the FTIR spectra of the HES phase after aqueous phase equilibration ([Eu^3+^]_HES_ = 0 g·dm^−3^).

Having ruled‐out speciation (i.e., decrease in the TOPO coordination) as an explanation to changes in Δ*H*_ex_ and Δ*S* *_ex_*, attention is now turned to HES solvent effects. To this end, the HES phase (*x*_TOPO_ = 0.5) after contact with an initial aqueous phase with Eu^3+^ concentration from 0 to 18 g·dm^−3^ (after extraction, the concentration in the HES varies from [Eu^3+^]_HES_ = 0 g·dm^−3^ to 35 g·dm^−3^, Table S11) was studied by Fourier transform infrared spectroscopy (FTIR) and ^31^P and ^1^H nuclear magnetic resonance (NMR). The full FTIR spectra are shown in Figure S9 and the regions specific to the P=O stretch band of TOPO and the C=O stretch band of decanoic acid are presented in Figure 3. To better evaluate the change with metal loading, the two‐dimensional correlation FTIR is available in Figure S10. The HES without metals displays the signature of intermolecular hydrogen‐bonding, with the disappearance of the free TOPO ligand band at 1155 cm^−1^ and the appearance of an intense band split at 1106 and 1130 cm^−1^. Conversely, the substitution of the carboxylic dimer by the weaker TOPO–decanoic acid adduct results in the shift of the C=O stretch band from 1709 cm^−1^ to 1715 cm^−1^. After Eu(NO_3_)_3_ extraction and coordination, the P=O stretch band is redshifted by up to 8 cm^−1^ (Figure S11) as expected when TOPO changes from HBA with decanoic acid to direct metal–ligand bonding. The shift upon complexation with Eu^3+^ is accompanied by a linear increase in the absorbance intensity due to the greater dipole moment of the P=O bond (greater ionic character). In turn, the absorbance of the carbonyl band of decanoic acid decreases linearly with [Eu^3+^] (Figure S11), albeit to a lesser extent. This decrease is mirrored in the absorbance of the OH stretch region of decanoic acid at approximately 2800 cm^−1^ (Figure S12). The 1738 cm^−1^ shoulder of the C=O stretch is absent without metal and grows with Eu(NO_3_)_3_ concentration, mirroring the loss of intensity at 1713 cm^−1^. The FTIR data are consistent with the weakening of the original hydrogen‐bond network in the HES following SX, with the breaking of the HBA‐HBD adduct and the release of loosely bound decanoic acid molecules to accommodate the Eu(NO_3_)_3_(TOPO)_3_ complex.

The ^31^P and ^1^H NMR full spectra of HES pure and after nitric acid and europium nitrate extraction are presented in Figures S13 and S14. The ^31^P NMR spectrum of TOPO, which was previously shown to be sensitive to the hydrogen bond strength [25], is shifted from 45.37 ppm in d_6_‐acetone [26] to ~51 ppm in the HES. After equilibration with the aqueous phase in the absence of Eu(NO_3_)_3_, the ^31^P resonances shift further downfield to ~53 and ~56 ppm, consistent with extraction of HNO_3_. Following europium extraction, the ^31^P signal becomes markedly broadened at low metal loading ([Eu^3+^]_HES_ = 3.5 g dm^−1^), with only a weak maximum remaining near ~57 ppm. At higher europium concentrations ([Eu^3+^]_HES_ = 35 g·dm^−1^), no ^31^P signal is detected, indicative of TOPO coordination to Eu^3+^ combined with paramagnetic relaxation effects. In addition, ^1^H NMR data show that the carboxylic acid proton shifts from 12.6 ppm in pure decanoic acid to 11.7 ppm in the HES, indicating hydrogen‐bond formation with TOPO. Upon nitric acid extraction, this signal moves upfield to 9.49 ppm, reflecting a modified hydrogen‐bonding environment. In contrast, Eu^3+^ extraction induces a progressive downfield shift of the decanoic acid proton (10.0 ppm at low metal loading and 13.7 ppm at higher concentrations), consistent with disruption of the TOPO–HBA interaction as TOPO preferentially coordinates Eu^3+^. To summarize, the complementary FTIR and NMR data demonstrate that Eu^3+^ coordination to the P=O group of TOPO progressively replaces TOPO–decanoic acid hydrogen bonding, leading to significant changes in the local structure of the HES.

## 3 Conclusion

This work provides a direct thermodynamic comparison of metal extraction in a conventional SX phase and in an HES using the same extractant. While both ITC and van’t Hoff analyses yield consistent thermodynamic trends, ITC offers a direct, model‐independent measurement of extraction enthalpy, making it particularly valuable for mechanistic interpretation and the rational design of solvent extraction systems. Overall, results for both systems indicate that SX in HES appears as an extension rather than a “revolution” of SX in organic diluents. However, significant differences are evident:

1. Although similar Δ*G*_ex_ were observed in both systems, europium partition was always higher in the HES system at the studied aqueous phase composition.
2. The decrease in the Δ*H*_ex_ observed at *T* = 298 K in ITC results (in comparison to conventional SX) is attributed to the competitive effect arising between metal–ligand and ligand–HBD interactions.
3. Speciation effects between systems are negligible and cannot explain the lower Δ*H*_ex_ and Δ*S*_ex_ values in HES.
4. The reduction of the Δ*S*_ex_ term in the HES relative to the conventional SX matches the expected effect of its higher TOPO concentration.

This study highlights both the opportunities and challenges in selective metal extraction using nonionic extractants. Further, its detailed thermodynamic characterization allows for an informed decision into the development of new extraction systems. The hydrogen‐bond interactions present may reduce the enthalpic term, counteracting the driving force for extraction. Yet they stabilize the extractant in the liquid phase, decreasing the entropic contribution Δ*S*_ex_ and allowing higher loadings and more efficient partitioning. Some questions remain open, including the effect of HBD type, HES composition, solute coextraction (e.g., water or acid), and other molecular interactions. Understanding these factors will be essential for tuning acid–ligand interactions, allowing the design of more efficient, stable, and highly selective HES formed with nonionic extractants.

## Conflicts of Interest

The authors declare no conflicts of interest.

## Supporting information

The authors have cited additional references within the Supporting Information [27, 28, 29, 30, 31, 32, 33, 34, 35, 36, 37, 38, 39, 40, 41, 42, 43, 44, 45, 46]. Data for this article, including ITC methodology, metal partition results, thermodynamic analysis, EXAFS, and spectroscopic results are available in the Supporting Information and/or at Zenodo at URL https://zenodo.org/communities/designsx/.
