# Supplementary material for: Identifying the Thermodynamic Driving Force of Metal Extraction by Hydrophobic Eutectic Solvents
Source: ChemSusChem. 2026 Jun 7;19(11):e70794. doi: 10.1002/cssc.70794 (PMC13242868; doi:10.1002/cssc.70794)
Supplement: Supplementary file 1 — The authors have cited additional references within the Supporting Information [27, 28, 29, 30, 31, 32, 33, 34, 35, 36, 37, 38, 39, 40, 41, 42, 43, 44, 45, 46]. Data for this article, including ITC methodology, metal partition results, thermodynamic analysis, EXAFS, and spectroscopic results are available in the Supporting Information and/or at Zenodo at URL https://zenodo.org/communities/designsx/. [file CSSC-19-e70794-s001.pdf]

Electronic Supporting Information

Identifying the thermodynamic driving force of metal extraction by hydrophobic eutectic solvents

Inês C.M. Vaz,†[a] Maísa Pinheiro,[a] Felipe Olea,[a] Luigi Cirillo,[b] Giorgia Mannucci,[b] Matteo Busato,[b] Paola D'Angelo,[b] Rui Santos,[c,d] Margarida Bastos,[d] Luís M.N.B.F. Santos,[d] João A.P. Coutinho,[a] and Nicolas Schaeffer†[a]

[a] CICECO – Aveiro Institute of Materials, Department of Chemistry, University of Aveiro, 3810-193 Aveiro, Portugal.

[b] Dipartimento di Chimica, Università degli Studi di Roma La Sapienza, P.le A. Moro 5, I-00185 Rome, Italy

[c] Analytik Jena GmbH at Centro de Investigação em Química (CIQUP), Faculdade de Ciências da Universidade do Porto, Rua Campo Alegre S/N, Porto, Portugal

[d] Centro de Investigação em Química (CIQUP), Institute of Molecular Sciences (IMS), Departamento de Química e Bioquímica, Faculdade de Ciências da Universidade do Porto, Rua do Campo Alegre S/N, Porto, Portugal

† Email: ines.vaz@ua.pt; nicolas.schaeffer@ua.pt

Contents

Experimental 3
Materials 3
Metal Quantification 3
Solvent Extraction (SX) Experiments 5
Thermodynamic Analysis 7
Isothermal titration calorimetry (ITC) 7
Measurement of Enthalpy of Metal Extraction 7
Heat Effects Simultaneous to Metal Extraction Experiments 12
Determination of Equilibrium Constants 14
Free Energy of Extraction 16
Determination of Enthalpy and Entropy of Extraction 18
Solute Co-extraction 21
Water 21
Nitric Acid 21
Extractant partition 22
Europium speciation 24
X-ray absorption spectroscopy (XAS) data collection and analysis 24
Spectroscopic measurements 28
FTIR 30
NMR 33
References 35

Experimental

Materials

Trioctylphosphine oxide (TOPO – 99.0 wt.% purity), decanoic acid (99.0 wt.% purity), nitric acid (65.0 wt.% purity) and toluene (99.5 wt.% purity) were purchased from Sigma Aldrich, europium(III) nitrate hydrate (99.99 wt.% purity) and sodium nitrate (99.5 wt.% purity) were obtained from Thermo scientific and HiMedia respectively. All chemicals were used as received. The water used in all experiments and methods is ultrapure deionized water, produced by reverse osmosis followed by Milli-Q purification (18.2 MΩ·cm).

The TOPO+Decanoic acid eutectic mixtures were prepared by mass using either an analytical balance (VWR®, LA 254i, ± 0.1 mg) or a precision balance (Mettler PM11-N, precision 0.1 g, maximum 11 kg) depending on the quantity prepared, followed by heating at 323 K under agitation for 60 min and stored in closed vials, but with no moisture control. The water content of the organic phases was measured by Karl-Fisher titration and is reported in the section "Solute Co-extraction". The phase diagram and other relevant physical-chemical properties of the eutectic mixture in this work were previously reported.[1]

Metal Quantification

Elemental Analysis by TXRF

The Eu(III) concentration in the aqueous phase was primarily determined by total reflection X-ray fluorescence (TXRF) using a Picofox S2 spectrometer (Bruker Nano) equipped with a molybdenum X-ray source, as this benchtop instrument was readily available in the laboratory. The voltage of the X-ray tube was 50 kV and the current 1000 µA. All carriers were first pretreated with 10 µL of silicon in isopropanol solution and dried at 353 K for 30 min. Ten microliters of the diluted sample containing Gallium as internal standard (final concentration of 10 ppm) was added onto a clean carrier and dried on a hot plate at 333 K for 15 min for analysis. An acquisition time of 180s was used for all samples.

Elemental Analysis by ICP-MS

Elemental analysis of samples was performed using a PlasmaQuant MS Elite S (Analytik Jena GmbH+Co. KG, Germany) inductively coupled plasma mass spectrometer (ICP-MS), equipped with a CETAC ASX-560 autosampler and ASXPress Plus injection valve for high-throughput, automated sample introduction. All measurements were conducted in a routine laboratory environment without cleanroom conditions. The ICP-MS was operated under optimized plasma and ion optics conditions as summarized in **Table S1**.

Table S1. PlasmaQuant MS Instrument Settings

Parameter	Specification
Plasma Gas Flow	10.5 $dm^3 \cdot min^{-1}$
Auxiliary Gas Flow	1.50 $dm^3 \cdot min^{-1}$
Sheath Gas Flow	0.00 $dm^3 \cdot min^{-1}$
Nebulizer Gas Flow	1.10 $dm^3 \cdot min^{-1}$
Sampling Depth	5.5 mm
Plasma RF Power	1.50 kW
Pump Rate	20 rpm – black/black PVC tubing (<1 $cm^3 \cdot min^{-1}$)
Stabilization Delay	10 s
iCRC Gas Settings	He (150 $cm^3 \cdot min^{-1}$): ^{153}Eu
Dwell Time	30 ms
Scans per Replicate	20 (peak hopping, 1 pt/peak)
Replicates	5
Sample Uptake Time	0 s (ASXPress Plus system)
Internal Standards	^{103}Rh and ^{193}Ir at 25 $\mu g \cdot dm^{-3}$ (interpolated correction)

Aqueous phase solutions obtained from solvent extraction experiments were diluted 20 to 25-fold prior to analysis. The europium stock solution used in the solvent extraction measurements was diluted 20,000-fold. Given the high metal concentration of some of the aqueous solutions of europium used for the spectrophotometric analysis, dilutions of up to 500,000-fold were performed. All the dilutions were prepared in 1% (v/v) HNO_3 (Blank). High-purity reagents were used throughout, including deionized water (>18.2 MΩ·cm, Milli-Q) and sub-boiled nitric acid (69%, Analytik Jena GmbH+Co. KG) to ensure trace-level accuracy and minimize contamination. External calibration was performed using matrix-matched standards prepared in 1% (v/v) HNO_3 in 50 cm^3 polypropylene tubes (Sarstedt, Germany). Single-element stock solutions (CertiPUR®, 1000 $mg \cdot dm^{-3}$ in 2–3% HNO_3) for Eu was diluted to prepare a five-point calibration curve at the following concentrations: 10, 25, 50, 100 and 250 $\mu g \cdot dm^{-3}$ for the target element (Eu).

Method Comparison

For method validation, the quantification of europium in an aqueous sample was done both by TXRF and ICP-MS. For TXRF analysis, a sample was diluted 100-fold and prepared in four independent replicates. For ICP-MS analysis, the sample was diluted 500- and 1000-fold, with each dilution corresponding to five independent preparations. The europium concentrations determined by TXRF and ICP-MS were 99 ± 3 $mg \cdot dm^{-3}$ and 99 ± 4 $mg \cdot dm^{-3}$, respectively. The

excellent agreement between the europium concentrations obtained by the two different methods confirming the accuracy and reliability of the methods used.

Solvent Extraction (SX) Experiments

Temperature-dependent extraction studies were conducted to determine the equilibrium constants, the Gibbs free energy of extraction as well as the derived enthalpy and entropy of extraction. All SX experiments were performed under controlled temperature conditions between 298 K and 328 K and prepared gravimetrically. Extractions were carried out in sextuplicate using a single-element europium solution prepared from europium oxide (Eu_2O_3) in 4.0 mol·dm^{-3} nitrate medium solution (0.1 mol·dm^{-3} HNO_3 + 3.9 mol·dm^{-3} $NaNO_3$). These conditions were selected to allow for comparison with a previously reported ITC study,[2] as well as to ensure the almost quantitative partition (D_{Eu} >> 100) of europium ions to the organic phases.

For all experiments, the initial europium concentration in the aqueous phase was fixed at [Eu^{3+}]= 16.7 mmol·dm^{-3}. Owing to the use of europium oxide for solution preparation, part of the nitric acid was consumed during dissolution, resulting in a slightly reduced effective acidity compared to nominal acid concentrations. The nitrate concentration in the aqueous phase was independently verified by UV–Vis spectroscopy using a PharmaSpec UV-1700 spectrophotometer (Shimadzu). Measurements were performed using a quartz cell with a 1 cm optical path length, monitoring the nitrate absorbance band at 301 nm. Quantification was achieved using a calibration curve established over the concentration range 0.14 – 0.19 mol·dm^{-3}, yielding an average nitrate concentration of 3.8 $\pm$ 0.3 mol·dm^{-3} in the samples. A constant organic-to-aqueous volumetric phase ratio (O:A) of 1:2 was employed unless otherwise specified. The partition of Eu(III) was studied in two organic phases, both containing TOPO as extractant. The first, referred to as "conventional SX", is a 200 mmol·dm^{-3} TOPO in toluene. The second, called "HES", is the TOPO+Decanoic acid HES phase with a TOPO molar fraction of x_{TOPO} = 0.5.

Europium partitioning from the aqueous phase to both conventional SX and HES organic phases respectively was investigated from 308 K to 328 K. All samples were either:

(i) agitated using a vortex mixer (Multi Reax, Heidolph) at 2000 rpm for 15 minutes, followed by 30 min of agitation in an Eppendorf Thermomixer C under temperature control (accuracy within ± 0.5 K). The samples were then left overnight in the Thermomixer C under temperature control only, to allow for phase disengagement.

(ii) rotated for 3 days in an orbital shaker (Trayster basic, IKA) and subsequently left to rest for 24 hours to allow for phase disengagement. All steps were carried out inside a home-built thermostatic cabinet with a temperature resolution of ± 0.1 K.

The aqueous phase was collected and diluted in 0.1 mol·dm^{-1} HCl prior to metal concentration analysis TXRF. Due to the elevated distribution of europium (D_{Eu}) values obtained, particularly in the HES system, ICP-MS was also employed for selected samples for more accurate measurements and reduce the error associated with the thermodynamic analysis (discussed further on). For these samples, the aqueous phase was collected and diluted in 1% (v/v) HNO_3. Both TXRF and ICP-MS metal quantification analysis details were described in the previous section.

The distribution of europium (D_{Eu}) in the systems was determined by mass balance according to:

$$D_{Eu} = [Eu]_{f,org} / [Eu]_{f,aq} \qquad - (1)$$

$$D_{Eu} = ([Eu]_{ini,aq} - [Eu]_{f,aq}) \times V_{aq} / ([Eu]_{f,aq} \times V_{org}) \qquad - (2)$$

Where [Eu] is the concentration of Eu(III) in mol·dm^{-3}, V is the phase volume in dm^3, and the subscripts org and aq indicate the organic and aqueous phase respectively, whilst the subscripts f and ini specify the timeframe – final and initial relative to the metal extraction.

The co-extraction of water and nitric acid was also verified and is reported in the section "Solute Co-extraction".

Thermodynamic Analysis

Isothermal titration calorimetry (ITC)

The calorimetric experiments were carried out using an isothermal titration calorimeter housed in a prototype 100 dm^3 water bath equipped with a high precision proportional-integral-derivative (PID) temperature controller developed and constructed at Lund University, Sweden. The isothermal titration calorimeter is a twin heat conduction instrument. For these experiments it was equipped with a 1 cm^3 hastealloy titration cell (Thermometric AB/TA) connected to a 71/2 digit Agilent nanovoltmeter (model 344420A) and to a computer running LABTERMO ITC software[3] for data acquisition and syringe-pump control.

The calorimeter was electrically calibrated in a previous study.[4] To evaluate the accuracy, the standard molar enthalpy of solution of pure propan-1-ol in water at infinite dilution was determined as recommended.[5] The obtained results (–10.10 ± 0.08) $kJ \cdot mol^{-1}$ are in good agreement with the recommended literature data (–10.16 ± 0.02 $kJ \cdot mol^{-1}$).[5] In addition, the partial molar enthalpy of propan-1-ol was measured up to a mole fraction of 0.08, showing good overlap with the data reported by Davis and Ham[6] and Tanaka et al.[7]

Measurement of Enthalpy of Metal Extraction

Prior to the start of each metal extraction experiment, the titration cell was loaded with the organic extractant phase, consisting of either 0.2 $mol \cdot dm^{-3}$ TOPO in toluene (SX system) or a TOPO–decanoic acid mixture (HES system, composition x_{TOPO} = 0.5), together with the corresponding aqueous phase composed of 0.1 $mol \cdot dm^{-3}$ nitric acid and 3.9 $mol \cdot dm^{-3}$ $NaNO_3$. The main titration cell tube, which houses the stirrer shaft, was wetted with the same liquid used as the upper phase. Injections into the cell were made using a modified 100 mm^3 gastight Hamilton syringe, through a stainless-steel capillary needle (inner diameter 0.15 mm), whose tip was immersed in the sample prior to the beginning of the experiment. After thermal equilibrium was reached, injections of the same aqueous solution containing europium, were made directly into the biphasic system. These solutions were prepared using different quantities of europium nitrate ($Eu(NO_3)_3$), depending on the desired level of europium. The exact quantity of europium was determined by TXRF. The reference cell was a solid stainless-steel cell in all ITC experiments.

All calorimetric measurements were performed at (298.15 ± 0.01) K and ambient pressure, under continuous stirring. The “slow titration mode” was used, where the calorimetric signal is always allowed to come back to the baseline before each new injection.

Interpretation of the calorimetric data was facilitated by designing experiments that ensured quantitative transport of the metal ion injected to the organic phase after each titrant addition. Experimental conditions were selected such that the extraction enthalpy could be verified to be independent of both the aqueous-phase metal-ion concentration or the organic-

to-aqueous (O:A) phase ratio, thereby confirming efficient phase partitioning equilibrium after each injection. In addition, injection volumes and the number of injections were chosen to maintain low metal loadings in the organic phase throughout the experiments, ensuring that the extraction stoichiometry remained constant within experimental uncertainty whilst providing sufficient heat production for accurate enthalpy determination. The experimental conditions are summarized in **Table S2**, including the aqueous-to-organic phase ratio, the concentration of the injected europium solution, the injection volume, the number of injections, as well as the resulting metal concentrations in the organic phase and the corresponding degree of ligand loading.

Table S2. Experimental conditions used for the ITC extraction experiments. Summary of the organic-to-aqueous (O:A) phase ratio, europium concentration in the injected aqueous phase ($[Eu^{3+}]_{aq,inj}$), injection volume (V_{inj}), number of injections (n_{inj}), resulting europium concentration in the organic phase after each injection ($[\Delta Eu^{3+}]_{org,i}$) and at the end of the experiment ($\sum_{i=1}^{n_{inj}}[\Delta Eu^{3+}]_{org,i}$), and the corresponding degree of ligand loading for both SX and HES systems

O:A ratio	$[Eu^{3+}]_{aq,inj}$ / mM	V_{inj} / mm^3	n_{inj}	$[\Delta Eu^{3+}]_{org,i}$ [a]/ mM	$\sum_{i=1}^{n_{inj}}[\Delta Eu^{3+}]_{org,i}$ [b]/ mM	$TOPO_{load,i}$ [c]/ %	$\sum_{i=1}^{n_{inj}} TOPO_{load,i}$ [d]/%
Organic Phase: 0.2 mol·dm^{-3} TOPO in toluene − SX system							
1:2	116	4.98	4	1.91	7.65	2.9	11
1:2	216	4.98	6	3.58	21.5	5.4	32
1:2	319	4.98	6	5.28	31.7	7.9	47
Organic Phase: x_{TOPO} = 0.5[e] TOPO-decanoic acid − HES system							
2:1	319	4.98	7	2.64	18.5	0.51	3.6
7:1	319	4.98	7	2.27	15.9	0.44	3.0

[a] europium concentration in the organic phase calculated after each injection and partition;

[b] total europium concentration in the organic phase at the end of the experiment;

[c] ligand loading per injection defined as $TOPO_{load,i} = 100 \times 3 \times [Eu^{3+}]_{org,i}/[TOPO]_{org}$;

[d] total ligand loading at the end of the experiment defined as $TOPO_{load,i} = 100 \times 3 \times \sum_{i=1}^{n_{inj}}[\Delta Eu^{3+}]_{org,i} / [TOPO]_{org}$;

[e] concentration estimated of TOPO in the HES system at x_{TOPO} = 0.5 is |TOPO|≈1.56 mol·dm^{-3}; all TOPO in the HES was considered as "free" (unbound).

The calorimetric signal obtained consists in heat flow as a function of time, with each injection producing a distinct peak. To correct for the instrumental time delay and to resolve the true real-time heat evolution in the cell, the recorded calorimetric signal was corrected using the Tian equation:

$$\frac{\mathrm{d}Q}{\mathrm{d}t} = \varepsilon \left(U + \tau \frac{\mathrm{d}U}{\mathrm{d}t} \right) \quad \text{- (3)}$$

The calorimetric data were analysed by integrating the individual peaks to obtain the heat exchanged per injection, q. In the case of $Eu(NO_3)_3$ extraction by TOPO, regardless of the organic phase used, the recorded peaks were exothermic. The enthalpy change associated with the extraction process was therefore calculated according to:

$$\Delta H = -\, q/\Delta n \quad \text{- (4)}$$

where the Δn term represents the moles of europium injected/partitioned per injection. (In this work, due to the high partitions verified for the SX systems being studied, the difference between considering the injected or partitioned europium, in determination of the enthalpy of extraction is negligible.)

Additionally, the reproducibility of achieving equilibrium conditions and ensuring efficient phase partitioning after each injection was confirmed by the absence of statistically significant differences between the extraction enthalpy values obtained by two methods, as reported in **Table S3**. Method (i) consists in calculating the average enthalpy from individual peak integrations divided by the amount of europium injected/partitioned, whereas method (ii) is based on the slope of the cumulative heat as a function of the total number of moles of metal injected/partitioned. Representative ITC thermograms and corresponding cumulative heat plots are shown in **Figures S1** to **S3**.

For the HES system, the experimentally conditions were previously optimized so that the enthalpy values obtained using the two methods and the different aqueous to organic phase ratios were essentially indistinguishable. For the conventional SX system, larger differences between both methods were observed, although they remain indistinguishable within the statistical threshold of $\pm\, 2 \cdot \mathrm{SD}/\sqrt{n}$. For the conventional SX system, a small relation with the concentration of the europium in the aqueous solution injected might also be present despite being within the limits of the uncertainty of the results. This is probably due to the higher loading of the ligand (see **Table S2**) and is also noticeable from the fact that for the injection of europium solutions of 216 and 319 $\mathrm{mmol \cdot dm^{-3}}$, the relation between cumulative heat and the quantity of europium partitioned deviates slightly from a linear relation, being better fitted by a second-degree polynomial.

Table S3. Summary of extraction enthalpies determined by ITC, obtained either from the slope of the cumulative heat versus total moles of europium injected/partitioned or from the average of the individual peak integrations, together with the experimental conditions: organic-to-aqueous (O:A) phase ratio, europium concentration in the aqueous phase injected [Eu^{3+}], injection volume (V_{inj}), and number of injections (n_{inj})

**O:A ratio**	**[Eu^{3+}] / mmol·dm^{-3}**	**V_{inj} / mm^3**	**n_{inj}**	**Δ*H* (slope) / kJ·mol^{-1}**	**Δ*H* (average) / kJ·mol^{-1}**	$\frac{2\sigma}{\sqrt{n_{inj}}}$
Organic Phase: 0.2 mol·dm^{-3} TOPO in toluene − SX system[a]						
1:2	116 (148)	4.98	4	−39.0 (−30.5)	−38.7 (−30.3)	0.8
1:2	216 (296)	4.98	6	−38[b] (−28)[b]	−35 (−26)	2
1:2	319 (450)	4.98	6	−38[b] (−27)[b]	−33 (−23)	3
Organic Phase: x_{TOPO} = 0.5 TOPO-decanoic acid − HES system						
2:1	319	4.98	7	−31	−30	1
7:1	319	4.98	7	−32.0	−31.8	0.7

[a] values in parentheses represent the revised results if the europium nitrate salt used to prepare the solutions is assumed as anhydrous.
[b] the data was better described by a second-order polynomial, the coefficient of the linear term was used to represent the average molar enthalpy of extraction.

The europium concentrations in the aqueous solutions, determined by TXRF was used for the calculation of the enthalpy values. They were consistent with the europium nitrate salt used for solution preparation being in the hexahydrate form: $Eu(NO_3)_3{\cdot}6H_2O$. Nevertheless, if the europium nitrate salt used for solution preparation had been assumed to be anhydrous, $Eu(NO_3)_3$, rather than determining the actual europium content of the solutions as we did, the extraction enthalpy values obtained for the SX system (see values in parenthesis in **Table S3**) would agree with literature values[2] within experimental uncertainty, despite the use of less favourable aqueous-to-organic phase ratios and higher europium concentrations in the aqueous phase than those reported. This clearly demonstrates the adequacy of the experimental conditions employed.

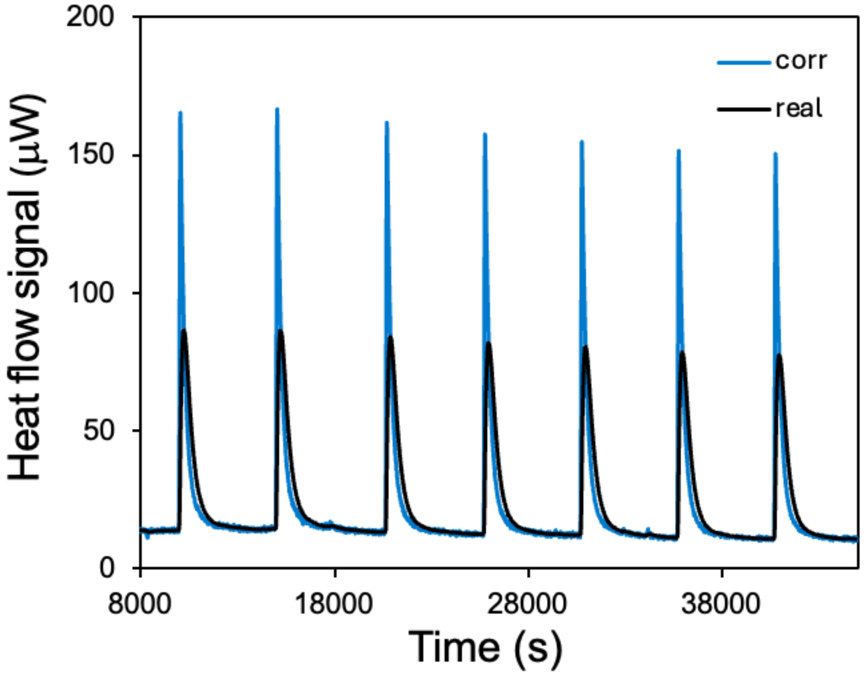

Figure S1. ITC thermogram of the injection of an aqueous solution of $[Eu^{3+}]$ = 319 mmol·dm^{-3}, $[HNO_3]$ = 0.1 mol·dm^{-3} and $[NaNO_3]$ = 3.9 mol·dm^{-3} in a system constituted by TOPO-decanoic acid HES (O:A = 1:2). Raw data (black line); corrected data using the Tian equation (blue line).

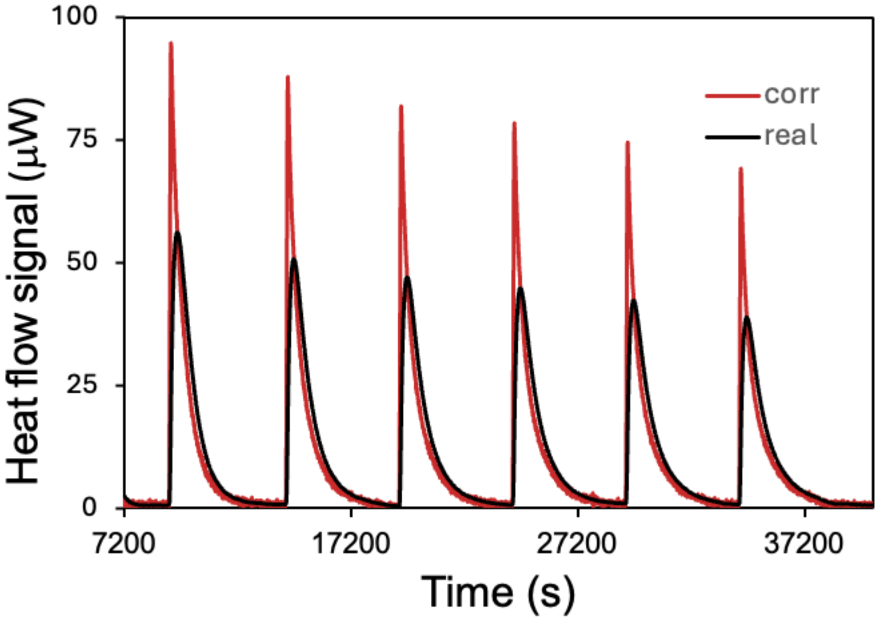

Figure S2. ITC thermograms of the injection of an aqueous solution of $[Eu^{3+}]$ = 319 mmol·dm^{-3}, $[HNO_3]$ = 0.1 mol·dm^{-3} and $[NaNO_3]$ = 3.9 mol·dm^{-3} in a system constituted by 0.2 mol·dm^{-3} TOPO in toluene (O:A = 1:2). Raw data (black line); corrected data using the Tian equation (red line).

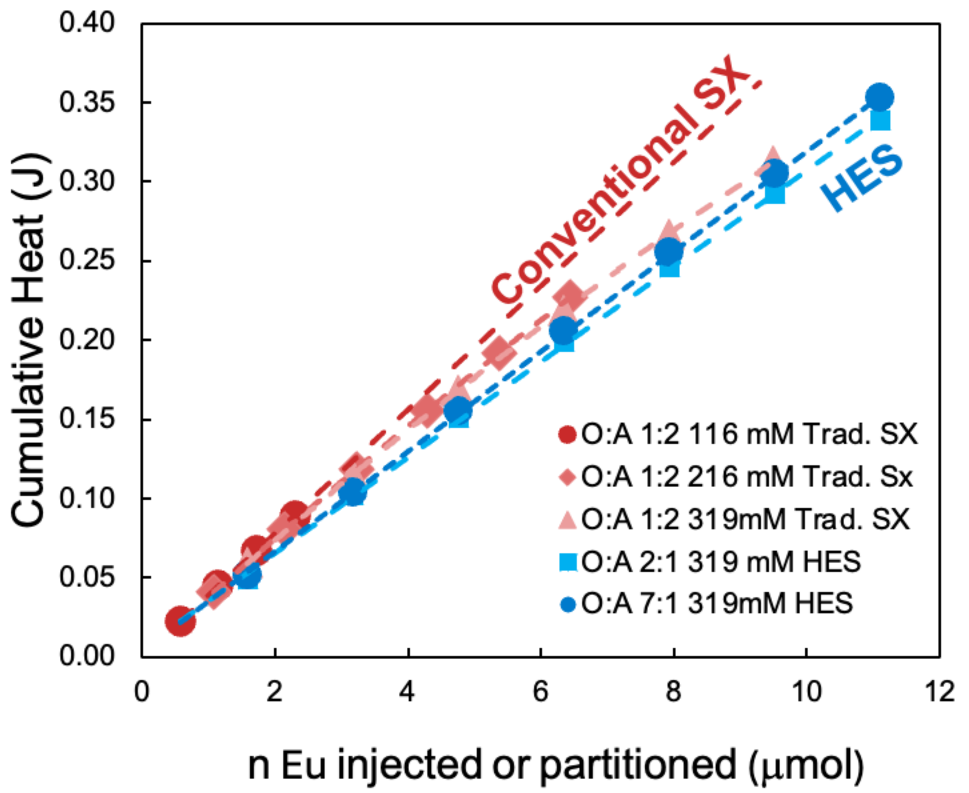

Figure S3. Cumulative heat plots for the experiments of extraction of europium by TOPO in conventional SX or HES.

<u>**Heat Effects Simultaneous to Metal Extraction Experiments**</u>

To successfully determine the enthalpy of extraction, a careful experimental design was implemented to ensure that no associated heat effects could introduce systematic deviations in the measured heat of extraction. In addition to the enthalpy associated with metal extraction, several other heat effects can occur during the experiments, including heat from nitric acid extraction, aqueous phase mixing, and metal dilution. These contributions must be minimized (or at least quantified and accounted) to ensure an accurate determination of the metal extraction enthalpy.

A critical step in minimizing these unwanted heat effects is the pre-saturation of both aqueous and organic phases with the other phase prior to the experiment at the working temperature of the calorimeter. Saturating the phases serves several important purposes:

- <u>Reduces equilibration time in the calorimeter before the beginning of the experiment</u>: Every biphasic system has some degree of miscibility. Small differences in the miscibility of the organic and aqueous components at different temperatures will slightly shift the equilibrium and probably cause a heat effect. Pre-saturation of the phases outside the calorimeter at the measurement temperature ensures that most of the equilibrium adjustments occur before the experiment begins, minimizing the time required for the system to reach equilibrium inside the calorimeter cell. While minor adjustments of

equilibrium will still occur due to small temperature differences, these are minimal and reduce calorimeter baseline drift during titration experiments.

- Minimizes spurious heat peaks unrelated to metal extraction: Pre-equilibrating the phases largely eliminates heat effects from phase transfer of water, nitric acid, or other minor components, allowing the calorimetric signal to reflect primarily the enthalpy of metal transfer. If the phases are accurately saturated, contributions from nitric acid extraction or aqueous phase mixing are negligible.

As a validation that the biphasic system is well equilibrated, blank injections with the aqueous solution of 0.1 mol·dm^{-3} HNO_3 with 3.9 mol·dm^{-3} $NaNO_3$ were carried out. These injections were carried out in several configurations: (i) into the aqueous phase itself, (ii) into a biphasic system constituted by the aqueous phase and toluene, (iii) into a biphasic system constituted by the aqueous phase and 0.2 mol·dm^{-3} of TOPO in toluene, and (iv) into a biphasic system constituted by the aqueous phase and the HES system. In all cases, even in the configurations with the organic phases containing TOPO, negligible heat effects were observed using the adopted experimental procedure.

Previous studies indicate that TOPO can extract HNO_3 as a 1:1 or 1:2 adduct depending on the HNO_3 concentration. However, only the 1:1 complex is expected under the experimental conditions used.[8–10] The ΔH_{ex} of HNO_3 extraction by TOPO was reported as −47.0 kJ mol^{-1}.[2] Nevertheless, the absence of significant heat signals in the blank experiments performed demonstrates the effectiveness of the phase saturation method, confirming that the phases were properly equilibrated prior to titration with the aqueous phase containing europium.

As the heat effect of the blank is negligible, the next relevant contribution could be the metal dilution in the aqueous phase. However, given that the higher magnitude of the metal partition in our experiments, the heat associated with metal dilution in the aqueous phase does not contribute meaningfully to the measured extraction enthalpy and therefore does not need to be considered separately.

Nonetheless, metal dilution experiments were performed to verify whether significant systematic deviations could arise from this assumption. The experiments consisted of injecting the aqueous europium solution into (i) the aqueous phase alone, (ii) the aqueous phase pre-saturated with toluene, and (iii) the aqueous phase pre-saturated with the HES extractant system. Two initial europium concentrations, [Eu^{3+}] = 319 mmol·dm^{-3} and [Eu^{3+}] = 225 mmol·dm^{-3}, corresponding to those used in the metal extraction experiments, were tested. The enthalpies of dilution were measured as −1.1 ± 0.1 kJ·mol^{-1} and −0.7 ± 0.1 kJ·mol^{-1}, respectively, with no meaningful differences observed between dilution into saturated or non-saturated aqueous phases. Given the magnitude of these heats and the uncertainties of the metal extraction measurements, it was considered that heat of metal dilution does not introduce significant deviations in the enthalpy of extraction.

It should be noted that metal dilution into the organic phase was not investigated. Under the experimental conditions, the extraction enthalpy (ΔH_{ex}) reflects the total enthalpy change for the transfer of metal ions from the aqueous phase to the organic phase, and any subsequent dilution (or better, concentration) within the organic phase is inherently included in this measurement. While studying dilution in the organic phase could provide additional insights into ΔH_{ex} and may be explored in future works, it does not constitute a separate process from the extraction enthalpy.

Determination of Equilibrium Constants

The extraction equilibrium of lanthanides by neutral phosphine oxide ligands such as TOPO is determined by a solvation mechanism, which involves the extraction and solubilisation of cation and its counter-ion in the organic phase by the neutral extractant. As such, the extraction of Eu^{3+} by TOPO in nitrate media can be expressed as follows:[2,11]

$$Eu^{3+}_{(aq)} + 3NO_3^-{}_{(aq)} + 3TOPO_{(org)} \rightleftharpoons Eu(NO_3)_3(TOPO)_{3(org)} \qquad -(5)$$

where (aq) and (org) represent the aqueous and organic phases, respectively. From equation (5), the extraction equilibrium constant (K_{ex}) is obtained as follows:

$$K_{ex} = \frac{[Eu(NO_3)_3(TOPO)_3]_{(org)}}{[Eu^{3+}]_{(aq)} \cdot [NO_3^-]^3_{(aq)} \cdot [TOPO]^3_{(org)}} \qquad -(6)$$

To take into consideration the expected aqueous phase speciation of europium in aqueous phases containing 4.0 mol·dm^{-3} nitrate, namely Eu^{3+}, $Eu(NO_3)^{2+}$, and $Eu(NO_3)_2^+$, the distribution coefficient D_{Eu} in equation (1) can be re-written as:

$$D_{Eu} = \frac{[Eu(NO_3)_3(TOPO)_3]_{(org)}}{[Eu^{3+}]_{(aq)} + [Eu(NO_3)^{2+}]_{(aq)} + [Eu(NO_3)_2^+]_{(aq)}} \qquad -(7)$$

or, equivalently, using the formation constants β_i for the aqueous nitrate complexes:

$$D_{Eu} = \frac{[Eu(NO_3)_3(TOPO)_3]_{(org)}}{[Eu^{3+}](1 + \sum \beta_i [NO_3{}^-]^i)} \qquad -(8)$$

where $\beta_{101} = 1.09 \pm 0.03$ and $\beta_{102} = 0.11 \pm 0.01$ are the stability constants for the $Eu(NO_3)^{2+}$ and $Eu(NO_3)_2^+$ complexes, respectively, as calculated by Grimes and coworkers.[2] Including equation (8) into the expression for the equilibrium constant (equation 6) and rearranging, the equilibrium constant can now be expressed as:

$$K_{ex} = \frac{D_{Eu}\left(1 + \sum \beta_i [\mathrm{NO_3^-}]_{\mathrm{aq}}{}^i\right)}{[NO_3^-]^3_{(aq)} \cdot [TOPO]^3_{(org)}} \qquad -(9)$$

The aqueous phase in this study contains a high ionic strength of approximately 4.0 mol·dm^{-3}. At such concentrations, non-ideal behaviour cannot be neglected, and activities must be considered instead of nominal concentrations ($a_{\mathrm{NO_3^-}} = \gamma_{\pm}[\mathrm{NO_3^-}]_{\mathrm{aq}}$). Therefore, in this work, the activities of the aqueous species were approximated by introducing a mean activity

coefficient ($\gamma_{\pm}$) for nitrate. For simplicity, and given that $NaNO_3$ is the dominant electrolyte, the mean activity coefficient corresponding to 4.5 mol·kg^{-1} $NaNO_3$ (at T = 298 K) reported by Hamer and Wu[12] ($\gamma_{\pm}$ = 0.396) was used as an approximation for the ionic mixture. For conventional SX systems using TOPO in toluene, the organic phase was assumed to behave ideally. That is, the activity of TOPO in toluene was taken as equal to its concentration: $a_{\mathrm{TOPO}} \approx [\mathrm{TOPO}]_{\mathrm{org}}$. Accordingly, equation 9 can now be re-written to reflect the calculation of equilibrium constant for the extraction of Eu^{3+} by TOPO in nitrate media to the conventional SX system:

$$K_{ex} = \frac{D_{Eu}\left(1+\sum \beta_i\left(\gamma_{\pm}[\mathrm{NO_3^-}]_{\mathrm{aq}}\right)^i\right)}{\left(\gamma_{\pm}[\mathrm{NO_3^-}]_{\mathrm{aq}}\right)^3 \cdot [TOPO]^3_{(org)}} \quad - (10)$$

Nevertheless, for the extraction system involving TOPO in a eutectic mixture with decanoic acid, the rigorous definition of free TOPO in the organic phase is complex. Relative to classical SX, a considerable but difficult to determine fraction of TOPO in the HES is involved in hydrogen-bonding interactions with decanoic acid ([TOPO·(Decanoic acid)$_n$]). Formally, the concentration of free TOPO could be approximated as:

$$[\mathrm{TOPO}]_{\mathrm{org}} \sim [\mathrm{TOPO}]_{\mathrm{tot}} - [(\mathrm{Decanoic\ acid})n\cdot\mathrm{TOPO}]_{\mathrm{org}} \quad - (11)$$

Accordingly, as a first approach, the non-ideality of the organic phase was taken into account through the activity coefficient of TOPO in the TOPO–decanoic acid mixture (γ_{TOPO}), calculated using the conductor-like screening model for real solvents (COSMO-RS) model[13,14] for a mole fraction x_{TOPO}= 0.5 at different temperatures. The effective activity of TOPO was thus defined as: $a_{\mathrm{TOPO}} = \gamma_{\mathrm{TOPO}}\cdot[\mathrm{TOPO}]_{\mathrm{Tot,org}}$. Substituting this activity into the equilibrium constant equation, the expression for Eu^{3+} extraction retains the same form as for TOPO in toluene, but now reflects the non-ideality of the organic phase of the HES system:

$$K_{ex} = \frac{D_{Eu}\left(1+\sum \beta_i\left(\gamma_{\pm}[\mathrm{NO_3^-}]_{\mathrm{aq}}\right)^i\right)}{\left(\gamma_{\pm}[\mathrm{NO_3^-}]_{\mathrm{aq}}\right)^3 \cdot \left(\gamma_{\mathrm{TOPO}}\cdot[\mathrm{TOPO}]_{\mathrm{Tot,org}}\right)^3} \quad - (12)$$

In this way, and despite being a simplification, the COSMO-RS approach accounts for its effective availability for europium extraction, ensuring a thermodynamically consistent evaluation of the equilibrium constant.

The equimolar approximation implemented in COSMO-RS was applied using COSMOtherm (version 25.0.0).[15] The individual precursors were optimized separately and subsequently combined at an equimolar composition (molar fraction of 0.5 for each component) to calculate the TOPO activity coefficient in the mixture (γ_{TOPO}) over the investigated temperature range. Molecular optimizations were performed using Turbomole® (TmoleX version 4.5)[16] at the density functional theory level, employing the generalized gradient approximation (GGA) Becke–Perdew functional (BP86)[17] with D4 dispersion correction.[18] A triple-zeta valence basis set with polarization and diffuse functions (def2-TZVPD)[19] was used

together with a FINE COSMO cavity.[20] Conformational effects were included by employing multiple representative conformers for each precursor in the COSMO-RS calculations. Given that activity coefficients and concentration-based equilibria were explicitly considered, extraction constants derived from mass-balance concentration data offer a more physically meaningful description for systems containing associating extractants such as TOPO + decanoic acid mixtures, as they account for deviations between analytical and effective extractant concentrations that are not captured by the Kex obtained through slope analysis.

Free Energy of Extraction

Having determined the equilibrium constants for extraction of europium by TOPO in the conventional SX system and in the HES system (**Table S4**), the Gibbs energy of extraction (ΔG_{ex}) can now be obtained from the extraction equilibrium constants following the equation:

$$\Delta G_{ex} = -RT \cdot \ln(K_{ex}) \qquad - (13)$$

where R is the gas constant (R = 8.314462618 $J \cdot mol^{-1} \cdot K^{-1}$) and the T is the absolute temperature in Kelvin (results summarized in **Table S4**).

In **Figure S4** the temperature dependence of europium extraction is evaluated. In **Figure S4A** the distribution coefficient (D_{Eu}) and the extraction equilibrium constant (K_{ex}) are plotted as a function of temperature, using a logarithmic scale on the y-axis to emphasize relative changes. This representation provides complementary information: D_{Eu} describes the practical distribution of europium between phases, whereas K_{ex} captures the intrinsic thermodynamic tendency for extraction after correction for aqueous speciation and non-ideality. The fact that both quantities exhibit essentially parallel trends over the investigated temperature range indicates that the apparent extraction behaviour and the intrinsic equilibrium constant are governed by the same underlying thermodynamic driving force. It is also evident that extraction partition is always superior when the organic phase is constituted by the HES system.

In **Figure S4B**, the Gibbs energy of extraction (ΔG_{ex}), calculated from the equilibrium constants, is presented as a function of temperature, providing a direct thermodynamic perspective on the temperature dependence of the europium extraction process. For both type of organic phases used, HES and conventional SX systems, the Gibbs energy of extraction of europium seems to become less negative with increasing temperature. Nevertheless, in liquid-liquid extractions performed with the HES system, experiments performed at higher temperatures, T = 318 K and T = 328 K, suggest that there might be a different behaviour.

Table S4. Summary of Eu^{3+} extraction data. The table reports the experimental equilibrium aqueous concentrations of europium ($[Eu^{3+}]_{aq,eq}$), the distribution coefficients D_{Eu}, the TOPO activity coefficients in both organic phases (γ_{TOPO}), the effective TOPO activities (a_{TOPO}), the calculated equilibrium constants (K_{ex}), and the Gibbs energy of extraction (ΔG_{ex}) at the indicated temperatures

T /K	D_{Eu}	γ_{TOPO}	a_{TOPO}	K_{ex}	ΔG_{ex} / kJ·mol^{-1}
Organic Phase: 0.2 mol·dm^{-3} TOPO in toluene − SX system[a]					
298	702 ± 101	1	0.200	530 ± 76	−15.5 ± 0.4
308	359 ± 51	1	0.200	270 ± 38	−14.3 ± 0.4
318	312 ± 34	1	0.200	235 ± 25	−14.4 ± 0.3
Organic Phase: x_{TOPO} = 0.5 TOPO-decanoic acid − HES system[a]					
298	6857 ± 805	0.330	0.516	5008 ± 588	−21.1 ± 0.3
303	6335 ± 932	0.332	0.520	4622 ± 344	−21.3 ± 0.2
308	5486 ± 1951	0.335	0.524	4000 ± 1422	−21.2 ± 0.9
308	4655 ± 132	0.335	0.524	3394 ± 96	−20.82 ± 07
313	3488 ± 211	0.338	0.528	2541 ± 154	−20.4 ± 0.2
318	7270 ± 2903	0.341	0.533	5291 ± 2112	−23 ± 1
328	3952 ± 1173	0.346	0.541	2871 ± 852	−21.7 ± 0.8

[a] The initial europium concentration in the aqueous phase was, for all performed extractions, $[Eu^{3+}]_{aq,t=0}$ = 16.7 mmol·dm^{-3}. Temperature uncertainty = ± 0.5 K.

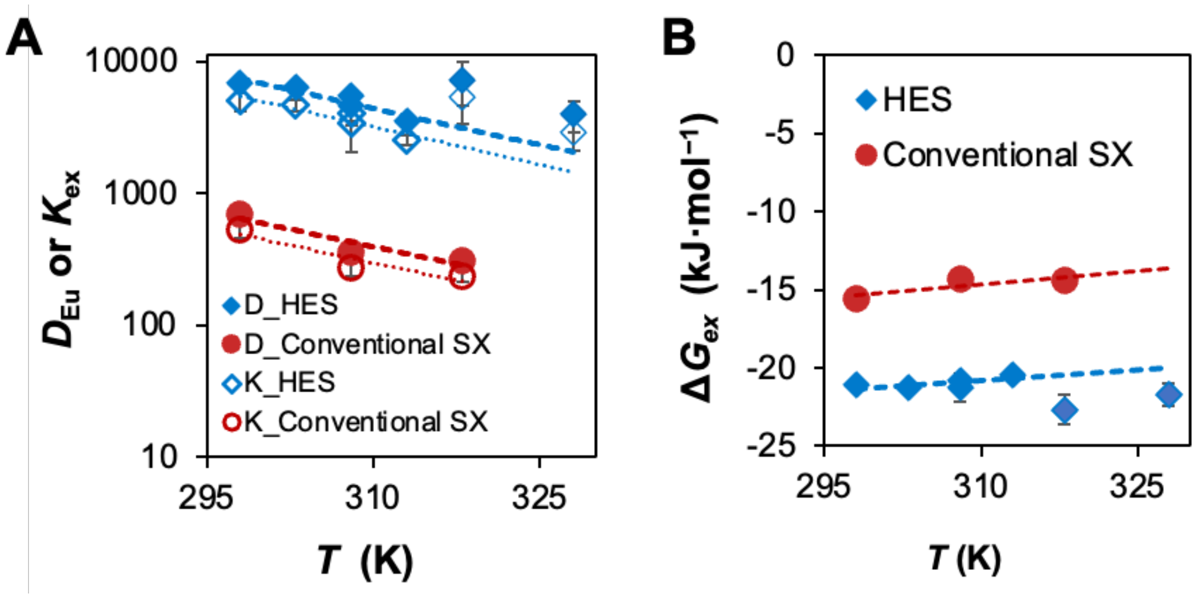

Figure S4. Temperature dependence of Eu^{3+} extraction for the investigated organic phases Conventional SX and HES. A) Distribution coefficients (D_{Eu}) and extraction equilibrium constants (K_{ex}) plotted as a function of temperature using a logarithmic scale on the y-axis. B) Gibbs energy of extraction (ΔG_{ex}) shown as a function of temperature. Lines are a guide to the eye.

Determination of Enthalpy and Entropy of Extraction

The enthalpy (ΔH_{ex}) and entropy (ΔS_{ex}) variation of the extraction can be deduced from the temperature dependence of K_{ex} as per the van't Hoff formalism:

$$\ln[K_{ex}(T)] = \left(-\frac{\Delta H_{ex}}{R}\right)\frac{1}{T} + \left(\frac{\Delta S_{ex}}{R}\right) \qquad - (14)$$

As part of the van't Hoff analysis, it was assumed that the extraction mechanism for europium partition in equation (5) remains constant within the studied temperature range, as suggested by the work of Suresh et al.[11] Under this assumption, ΔH_{ex} and ΔS_{ex} (listed in **Table S5**) were determined from the slope and intercept, respectively, of a linear plot of ln(K_{ex}) versus $1/T$ (**Figure S5**). In this scenario heat capacity (C_p) effects on the enthalpy and entropy were assumed to be constant over the temperature range investigated. For the HES system, the data at 318 K and 328 K were excluded from the analysis, as they appeared to deviate from the linear trend defined by the data between 298 K and 313 K (see **Figure S5**). Moreover, since the enthalpy of extraction (ΔH_{ex}) was independently determined by calorimetry at 298 K, these values can be combined with the Gibbs relation,

$$\Delta G_{ex} = \Delta H_{ex} - T\Delta S_{ex} \qquad - (15)$$

to calculate a more precise and accurate estimate of the entropy change at the specific temperature of the calorimetric measurement. This approach allows for a direct assessment of the thermodynamic driving forces under conditions where the enthalpy is accurately known, providing a complementary perspective to the van't Hoff analysis based on the

temperature dependence of the equilibrium constants. Together, these approaches provide a comparison and analysis between the extraction in a conventional solvent extraction system (TOPO in toluene) and the hydrogen-bonded eutectic system (TOPO–decanoic acid), revealing insights into its mechanisms.

Table S5. Summary of thermodynamic parameters of europium extraction at 298 K determined directly by van't Hoff analysis and by combination of calorimetric data and Gibbs relation

Org Ph.	T [a]/ K	ln(K_{ex}) [b]	ΔG_{ex} [b] / kJ·mol^{-1}	ΔH_{ex} / kJ·mol^{-1}	ΔS_{ex} / J·K^{-1}·mol^{-1}
			Van't Hoff Analysis [c]		
HES	298	8.5 ± 0.1	−21.1 ± 0.3	−36 ± 6	-50 ± 20
Conventional SX	298	6.3 ± 0.1	−15.5 ± 0. 3	−32 ± 13	−57 ± 43
			Calorimetric Data and Gibbs Relation [d]		
HES	298	8.5 ± 0.1	−21.1 ± 0.3	−31 ± 1	−33 ± 3
Conventional SX	298	6.3 ± 0.1	−15.5 ± 0.3	−38.4 ± 0.6	−77 ± 4

[a] Temperature uncertainty = ± 0.5 K.

[b] propagated from the uncertainty of the partition experiments, which was calculated as ± $2 \times \sigma/\sqrt{n},$ where $\sigma$is the standard deviation of the sextuplicate replicas and n = 6;

[c] Enthalpy and entropy is presented ± $2 \times \sigma/\sqrt{n}$, where $\sigma$is the standard deviation of the slope/intercept of ln K vs 1000/T and *n* is the number of temperatures;

[d] Enthalpy is presented ± $2 \times \sigma/\sqrt{n}$, where σ is the standard deviation of independent measurements (n = 2–3); entropy uncertainty is propagated from enthalpy and Gibbs free energy as $\pm 1000 \cdot \sqrt{u_{\Delta H}^2 + u_{\Delta G}^2}/T$.

In the temperature range considered, for both the conventional (0.2 mol·dm^{-3} TOPO in toluene) and HES (x_{TOPO} = 0.5, TOPO + decanoic acid) solvent extraction system, the ΔH_{ex} and ΔS_{ex} values obtained from van't Hoff analysis are in excellent agreement, within experimental uncertainty, with those derived from the calorimetric enthalpy combined with the Gibbs relation. This consistency suggests that europium extraction by TOPO is well described by a single dominant equilibrium. The extraction is exothermic and enthalpy-driven, the negative entropy of extraction reflecting an increase in molecular ordering upon formation of the solvated $Eu(NO_3)_3(TOPO)_3$ complex in the organic phase, fully consistent with the classical solvation mechanism of Eu^{3+} by TOPO in molecular diluents.

This should not be taken as a proof of van't Hoff analysis being valid for all HES systems. In fact, the presence of a less exothermic ΔH_{ex} obtained from van't Hoff analysis can be hypothesized to be present for larger temperature ranges or other HES systems due to the presence of secondary, temperature-dependent interactions, most notably hydrogen bonding between extractants as TOPO and hydrogen bond donors. As temperature increases, weakening of these interactions is expected to increase the fraction of free extractant available for metal complexation, leading to a smaller decrease in K_{ex} with temperature than would be expected. This effect can flatten the van't Hoff slope, resulting in a less exothermic enthalpy than would be expected for the extraction equilibrium alone, as observed in conventional solvent extraction systems.

Another important aspect is the uncertainty associated to the enthalpy and entropy of extraction of the metal complex, depending on the determination method used. Direct methods are expected to yield results with uncertainties approximately one order of magnitude smaller. Thus, to derive insights at the molecular level, direct measurements are always preferred as they provide a better resolution on the differences of both driving forces between systems.

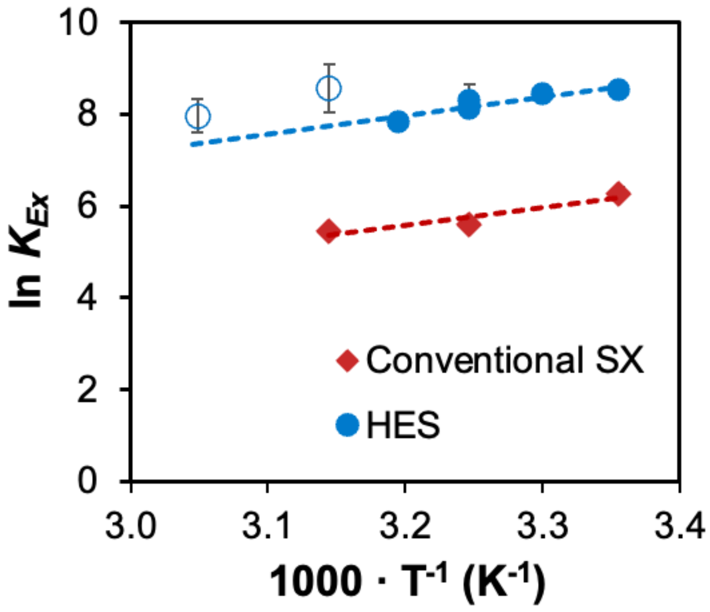

Figure S5. Van't Hoff plots for Eu^{3+} extraction in the conventional solvent extraction system (0.2 mol·dm^{-3} TOPO in toluene) and in the TOPO–decanoic acid HES (x_{TOPO} = 0.5). Symbols represent experimental values of ln(K_{ex}) as a function of inverse temperature (1/T), dashed lines correspond to linear regressions used to determine the enthalpy (ΔH_{ex}) and entropy (ΔS_{ex}) of extraction according to the Van't Hoff equation. Uncoloured blue circles were not considered in the van't Hoff fit.

Solute Co-extraction

Water

The water content in the HES and in the conventional SX phase after extraction was determined using coulometric Karl-Fisher titration, either with a Metrohm 831 KF Coulometer or a Metrohm Eco KF Titrator, using Hydranal™ solution from Honeywell. Since HNO_3 is incompatible with the titrating solution, the organic phase was first neutralized with TOA prior to titration. An excess of TOA (0.07 g to 0.10 g) was added to 0.25 g to 0.30 g of the HES sample, and the mixture was agitated at 298 K for 1 h before measurement. The water content of the sample was corrected by accounting for the water content of pure TOA, which was independently determined.

In this case, the organic-to-aqueous ratio (O:A) of the biphasic systems was 1:2 in mass. The water content of the HES mixture was found to be at most 0.23%, and no increase in water content after extraction was observed. For the conventional SX phase, water content was measured only after extraction and was found to be approximately 0.21%.

Nitric Acid

Nitric acid can be co-extracted by TOPO through the formation of neutral adducts according to the equilibrium:

$$HNO_3(aq) + n\ TOPO\ (org) \rightleftharpoons (HNO_3{\cdot}nTOPO)\ (org) \qquad - (16)$$

The co-extraction of nitric acid (HNO_3) was investigated under the same extraction conditions described in the Solvent Extraction Experiments section. For each experimental condition, three independent samples were collected for acid quantification. The co-extraction of HNO_3 was studied both in the absence and presence of metal cations (**Tables S6 and S7**). Nitric acid concentrations were determined using a Thermo Scientific OrionStar T940 automatic titrator, with titrations performed using a 0.01 $mol{\cdot}dm^{-3}$ NaOH solution.

The aqueous solutions containing europium were prepared with europium oxide salt. Therefore, in experiments conducted in the presence of europium, the aqueous solutions have a lower initial nitric acid concentration compared to metal-free systems. Considering the dissolution stoichiometry of Eu_2O_3 (1 mol Eu_2O_3 consuming 6 mol HNO_3), a europium concentration of 16.7 $mmol{\cdot}dm^{-3}$ corresponds to the consumption of approximately 50.1 $mmol{\cdot}dm^{-3}$ of nitric acid, a value consistent with the difference in the concentration of nitric acid present in both solutions. Despite this decrease in acidity, the remaining nitric acid concentration is expected to be sufficient to prevent the dissociation of decanoic acid.

Table S6. Distribution coefficients of nitric acid in the conventional SX system (0.2 mol·dm^{-3} TOPO in toluene) as a function of the temperature of extraction in the presence of europium and in its absence (O:A = 1:2).

T/K	298	308	318
**Extractions (in SX) in the Presence of Europium (III) -** $[HNO_3]_{ini,aq}$ = 31 mmol·dm^{-3}			
$[HNO_3]_{f,aq}$ / mmol·dm^{-3}	14 ± 1	15 ± 1	16 ± 1
$[HNO_3]_{f,org}$ / mmol·dm^{-3}	35 ± 1	31 ± 1	31 ± 1
D_{NO_3}	2.5 ± 0.2	2.0 ± 0.2	2.0 ± 0.2
**Extractions (in SX) without Europium (III) -** $[HNO_3]_{ini,aq}$ = 84 mmol·dm^{-3}			
$[HNO_3]_{f,aq}$ / mmol·dm^{-3}	29 ± 2	31 ± 1	31 ± 1
$[HNO_3]_{f,org}$ / mmol·dm^{-3}	110 ± 4	107 ± 1	107 ± 1
D_{NO_3}	3.8 ± 0.4	3.5 ± 0.1	3.5 ± 0.1

Table S7. Distribution coefficients of nitric acid in the TOPO-decanoic acid HES system as a function of the temperature of extraction in the presence of europium and in its absence (O:A = 1:2, x_{TOPO} = 0.5).

T/K	298	308	318	328
**Extractions (in HES) in the Presence of Europium (III) -** $[HNO_3]_{ini,aq}$ = 31 mmol·dm^{-3}				
$[HNO_3]_{f,aq}$ / mmol·dm^{-3}	6.3 ± 0.4	6.3 ± 0.3	6.5 ± 0.0	7.3 ± 0.3
$[HNO_3]_{f,org}$ / mmol·dm^{-3}	49.3 ± 0.7	49.3 ± 0.7	49.0 ± 0.0	47.3 ± 0.7
D_{NO_3}	7.8 ± 0.5	7.8 ± 0.5	7.5 ± 0.0	6.5 ± 0.4
**Extractions (in HES) without Europium (III) -** $[HNO_3]_{ini,aq}$ = 84 mmol·dm^{-3}				
$[HNO_3]_{f,aq}$ / mmol·dm^{-3}	16.3 ± 0.7	15.8 ± 0.7	16.5 ± 0.6	18 ± 1
$[HNO_3]_{f,org}$ / mmol·dm^{-3}	162 ± 1	163 ± 1	163 ± 1	158 ± 2
D_{NO_3}	9.9 ± 0.5	10.3 ± 0.5	9.8 ± 0.4	8.7 ± 0.7

Extractant partition

To evaluate the extractant partition to the aqueous phase, ^{1}H nuclear magnetic resonance (NMR) spectra were performed in aqueous phases obtained after solvent extraction using either TOPO in toluene (conventional SX) or a TOPO–decanoic acid HES as the organic phase. Extractions were performed without metal and at two different temperatures, 298 K and 318 K, under otherwise identical conditions. Deuterated water containing tetramethylsilane (TMS) was added using a coaxial insert as a reference. The ^{1}H nuclear magnetic resonance (NMR) spectra were performed on a Bruker AVANCE 300 MHz instrument equipped with a z-gradient double resonance probe. In **Figure S6** the ^{1}H spectrum of the aqueous phase obtained from

liquid-liquid extractions performed with HES at 298 K is represented. The only visible peak has a chemical shift of 4.37 ppm and is characteristic of D_2O, not being possible to identify any signal characteristic of TOPO or decanoic acid. All other spectra were identical independently of the organic phase or the temperature at which solvent extraction occurred.

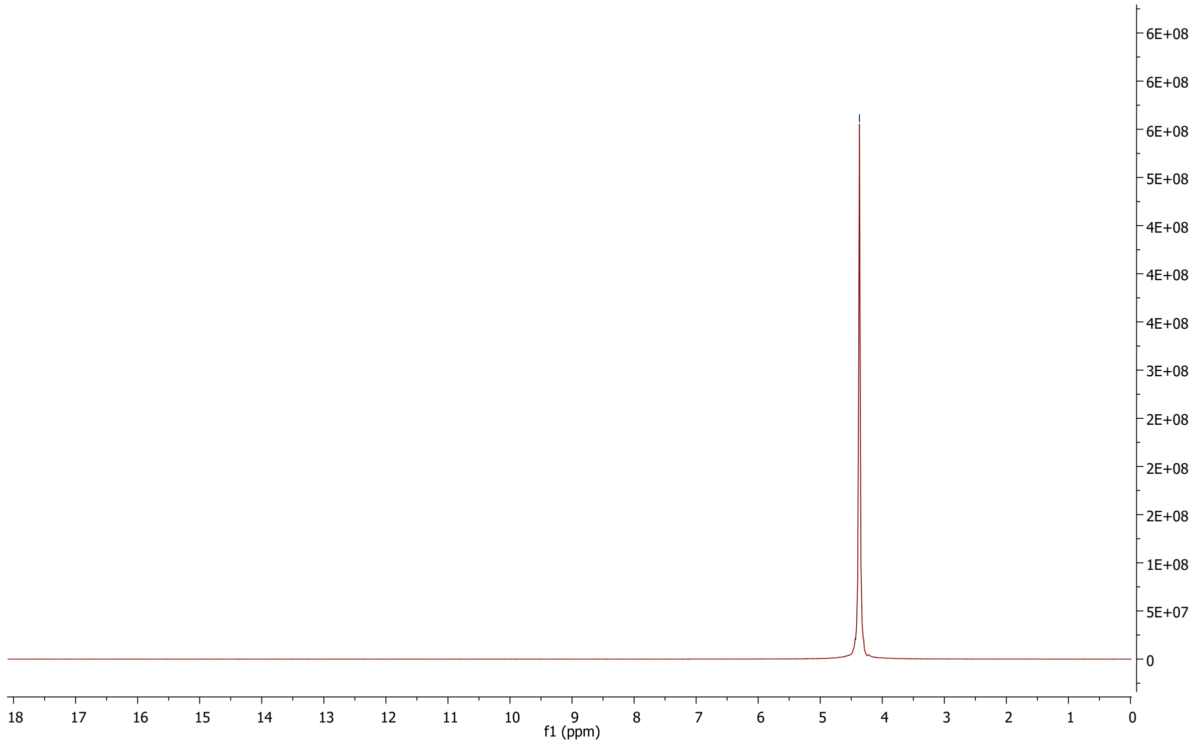

Figure S6. ^1H NMR spectrum (300 MHz, D_2O) of the aqueous phase (0.1 mol·dm^{-3} HNO_3, 3.9 mol·dm^{-3} $NaNO_3$) after liquid–liquid extractions performed with HES organic phase (x_{TOPO} = 0.5 TOPO-decanoic acid; O:A ratio = 1:2).

Europium speciation

X-ray absorption spectroscopy (XAS) data collection and analysis

XAS spectra above the Eu^{3+} L_3-edge were collected in transmission mode at the beamline BM23 of the European Synchrotron Radiation Facility (ESRF, Grenoble) as part of the proposal CH-7201 (https://doi.org/10.15151/ESRF-ES-1999194804). The data were collected with a Si(311) double-crystal monochromator with the second crystal detuned by 20% for harmonic rejection. The incident X-ray energy was calibrated using the K-edge energy of Fe foil (7.112 keV) due to its proximity with the L_3-edge energy of Eu (6.977 keV). The absorption spectra were measured from −150 eV to +600 eV relative to the europium L_3-edge energy. Three spectra were recorded for each sample and averaged. All samples were collected at room temperature in the liquid phase using capillaries of varying diameter from 0.8 mm to 3.0 mm depending on the solvent Eu^{3+} concentration. **Table S8** below summarises the experimental conditions of the analysed samples.

Table S8. Solvent extraction conditions (aqueous phase composition of 0.1 mol·dm^{-3} HNO_3 + 3.9 mol·dm^{-3} at T = 298 K and an O:A = 1:2) and final Eu^{3+} concentration in the organic phase for the samples analysed by XAS.

**Hydrophobic eutectic system (HES)**				
x_{TOPO}	$[Eu^{3+}]_{ini,aq}$ /mol·dm^{-3}	$[Eu^{3+}]_{f,org}$ /mol·dm^{-3}	Extraction %	D_{Eu}
0.3	0.152	0.106	34.93	1.07
0.3	0.071	0.066	46.46	1.74
0.3	0.025	0.031	60.34	3.04
0.5	0.152	0.224	73.69	5.60
0.5	0.071	0.139	98.19	108.8
0.5	0.025	0.051	99.82	1105
**Conventional SX (TOPO in dodecane with 5 vol% octanol)**				
[TOPO] /mol·dm^{-3}	$[Eu^{3+}]_{ini,aq}$ /mol·dm^{-3}	$[Eu^{3+}]_{f,org}$ /mol·dm^{-3}	Extraction %	D_{Eu}
0.2	0.152	0.051	16.68	0.40

To determine the local atomic structure of Eu^{3+} in the various organic phases, the analysis of the extended X-ray absorption fine structure (EXAFS) region was performed using the same strategy using the open source Larix software.[21] An R bkg of 1.7 was applied for the preliminary background subtraction, whilst the Fourier-transforms (FTs) of the absorption spectra were calculated in the 2.7–10.7 Å^{-1} k-range with a Kaiser Bessel k-window filter. Least-squares minimizations of the experimental data were performed using theoretical phase and amplitude functions calculated at the ab initio core-level using the FEFF8 code (embedded in Larix).[22]

The starting model was based on the crystal structure of tris(triethylphosphine oxide) europium tris(nitrato) (CCDC 866501) reported by Bowden et al.[23] As no differences were observed amongst the HES sample EXAFS spectra (see **Figure S7**), only the best-fit results for the conventional SX phase and x_{TOPO}= 0.5 for [Eu^{3+}]=0.05 mol·dm^{-3} are presented in **Figure S8**. Due to large number of two-body contributions at higher distances, the EXAFS data were fitted using only the Eu-O, Eu-N and Eu-P contributions while the higher frequency signals were not included in the calculations. Two structural parameters were optimized for each two-body signal, namely the distance *R*, and the Debye–Waller σ^2. The coordination number (N) was fixed to 3 for the Eu–N path, whilst the Eu–O *N* was varied in the range between 8 and 10 and Eu–P *N* was unbound.

Regarding the nonstructural parameters only the E_0 value defining the positions of the theoretical energy scale was varied, while the amplitude reduction factor was fixed at S_0^2 = 0.9. The larger ΔR values (the difference between the starting and optimized values) obtained during fitting of the Eu–O path is due to the presence of two Eu–O bond lengths in the model structure presenting a shorter Eu–TOPO interaction at 2.29 Å and longer Eu–NO_3^- at 2.52 Å in the crystal. However, modelling the EXAFS data with two distinct Eu-O single scattering contributions did not improve the fit. The best fit parameters obtained from the minimization procedure are summarised in **Tables S9** and **S10** for the HES and classical SX system, respectively.

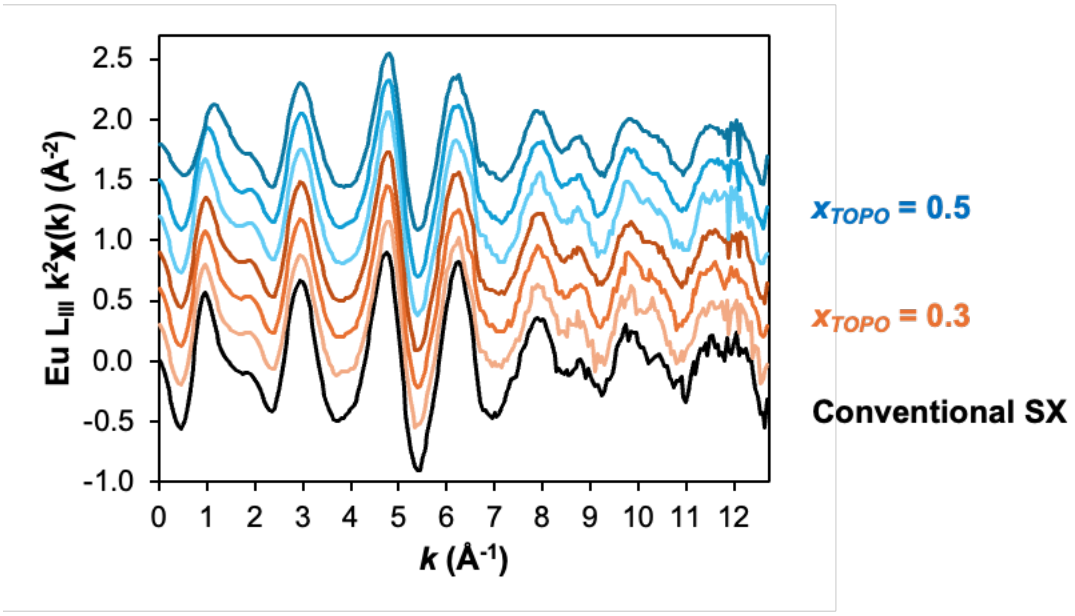

Figure S7. Eu^{3+} L_3-edge EXAFS experimental spectra of the conventional SX system (black lines), and in the HES phase for x_{TOPO} = 0.3 (orange lines) or 0.5 (blue lines) after contact with three initial aqueous phase $Eu(NO_3)_3$ concentrations (0.025, 0.071, 0.152 mol·dm^{-3}; colour code from light to dark reflects the concentration increase).

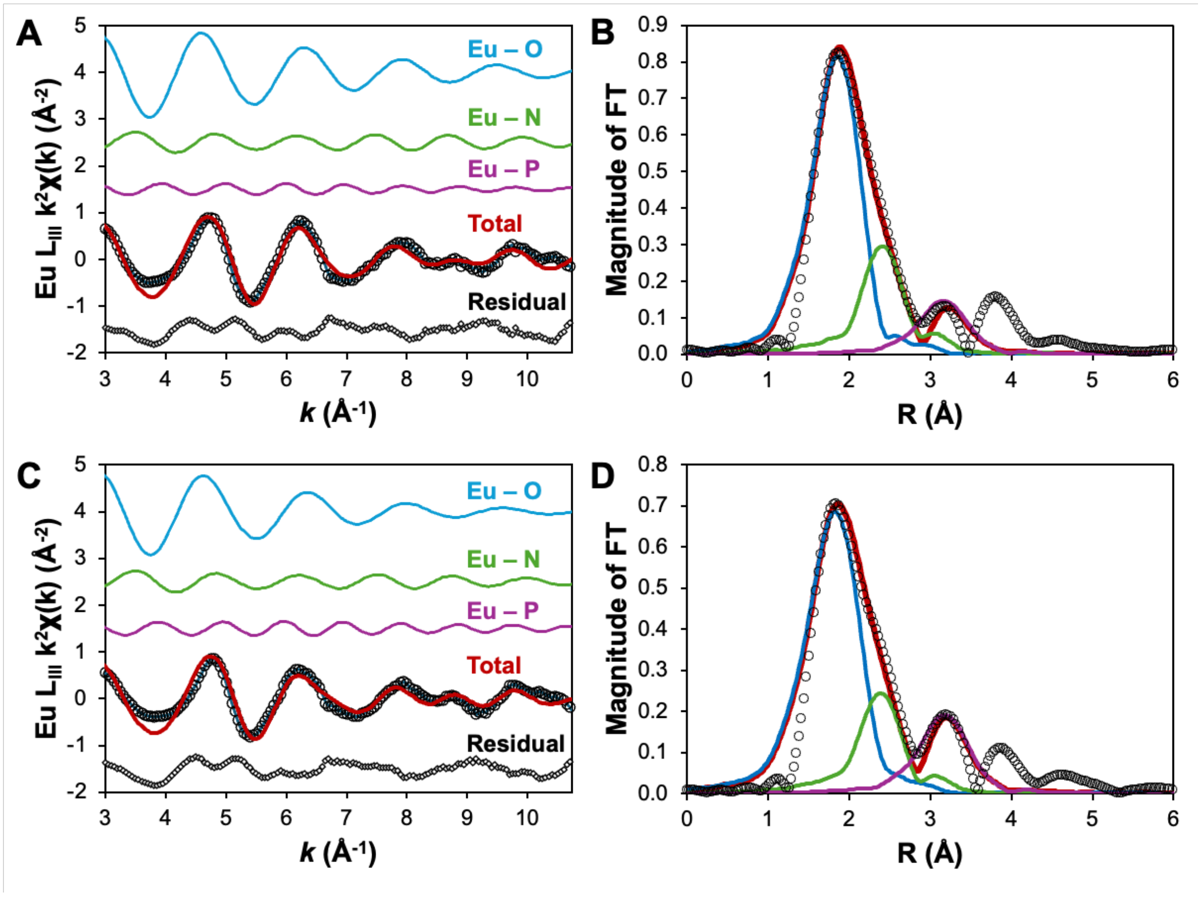

Figure S8. Best fit results for Eu^{3+} L_3-edge EXAFS (A and C) and corresponding non-phase shift corrected Fourier transform (B and D) in the conventional SX system (A and B) and in the HES phase for x_{TOPO} = 0.5 (C and D) for $[Eu^{3+}]_{f,org}$= 0.051 mol·dm^{-3}. The Eu–O, Eu–N, and Eu–P theoretical signals are shown in blue, green and purple respectively, whilst the total theoretical curve is presented in red superimposed with the experimental data (black circles) and the obtained residual (black diamonds).

Table S9. Structural and fitting parameters obtained from the EXAFS analysis of Eu^{3+} in the TOPO + Decanoic acid HES (x_{TOPO}= 0.5, $[Eu^{3+}]_{f,org}$= 0.051 mol·dm^{-3}). N is the coordination number, R is the two-body distance, σ^2 is the Debye-Waller factor, Δr is the difference between the crystallographic and EXAFS distance.

Paths	N	R (Å)	σ^2 ($Å^{-2}$)	ΔR	E_0 (eV)	S_0^2	Reduced χ^2	R-factor
Eu - O	9.1 ± 1.0	2.37 ± 0.04	0.013 ± 0.004	0.09	2.9	0.9	2.74	0.016
Eu - N	3.0[a]	2.93 ± 0.04	0.002 ± 0.004	-0.02	2.9	0.9	2.74	0.016
Eu - P	3.2 ± 1.0	3.78 ± 0.06	0.006 ± 0.010	0.04	2.9	0.9	2.74	0.016

[a] Eu – N coordination number was fixed to 3

Table S10. Structural and fitting parameters obtained from the EXAFS analysis of Eu^{3+} in the organic phase composed of 0.2 mol·dm^{-3} TOPO in dodecane with 5 vol.% n-octanol (x_{TOPO} = 0.5, $[Eu^{3+}]_{f,org}$ = 0.051 mol·dm^{-3}). N is the coordination number, R is the two-body distance, σ^2 is the Debye-Waller factor, Δr is the difference between the crystallographic and EXAFS distance.

Paths	*N*	*R* (Å)	σ^2 (Å^{-2})	*ΔR*	E_0 (eV)	S_0^2	Reduced χ^2	R-factor
Eu - O	8.3 ± 1.0	2.38 ± 0.02	0.009 ± 0.002	0.10	3.1	0.9	1.52	0.009
Eu - N	3.0[a]	2.94 ± 0.02	0.001 ± 0.001	-0.01	3.1	0.9	1.52	0.009
Eu - P	2.3 ± 1.0	3.76 ± 0.03	0.006 ± 0.007	0.02	3.1	0.9	1.52	0.009

[a] Eu – N coordination number was fixed to 3

Spectroscopic measurements

The objective of this spectroscopic study was to investigate the molecular interactions and structural reorganization within the TOPO–decanoic acid hydrophobic eutectic solvent (HES) induced by nitric acid and europium ion extraction. In particular, ^{1}H and ^{31}P nuclear magnetic resonance (NMR) spectroscopy, together with Fourier transform infrared (FTIR) spectroscopy, were employed to evaluate: (i) changes in the hydrogen-bonding interactions between TOPO and decanoic acid; (ii) the formation of TOPO–HNO_3 adducts; and (iii) the coordination of TOPO to Eu^{3+} and its impact on the organization of the HES phase.

FTIR and NMR spectra were recorded for the neat HES and for the HES phase after contact with an aqueous phase containing 0.1 mol·dm^{-3} HNO_3 and 3.9 mol·dm^{-3} $NaNO_3$, with increasing concentrations of $Eu(NO_3)_3 \cdot nH_2O$ ranging from 0 to 50 g·dm^{-3}. Liquid–liquid contact experiments were carried out at ambient temperature using an orbital shaker (Trayster basic, IKA) for 16 hr, followed by centrifugation at 10,000 rpm for 10 minutes (MPW-55 centrifuge) to ensure complete phase separation. After separation, the HES phase was recovered and analysed by NMR or FTIR without further treatment. The aqueous phases were diluted as required for europium quantification by ICP-MS, as described previously. The initial and final europium concentrations in the aqueous phase, as well as the corresponding concentrations in the HES phase, are reported in **Table S11.**

FTIR spectra were recorded on an JASCO FTIR-4X. Spectra were recorded from 400 to 4000 cm^{-1} with an attenuated total reflectance (ATR) in transmission mode with resolution of 4 cm^{-1} and 128 scan acquisition. Temperature was maintained constant during the acquisition using a heated accessory from Specac coupled to a PID temperature controller (model WEST 6100+). Two-dimensional correlation analysis of the spectra as a function of $Eu(NO_3)_3 \cdot nH_2O$ loading was performed using the 2D-Shige program written by Prof. Shigeaki Morita (Osaka Electro-Communication University) obtained from the webpage: https://sites.google.com/view/shigemorita/home/2dshige. ^{1}H and ^{31}P nuclear magnetic resonance (NMR) spectra were performed on a Bruker AVANCE 300 MHz instrument equipped with a z-gradient double resonance probe. The organic phases, prepared and loaded with Eu^{3+} under the same conditions as described, were analysed at 308 K. Deuterated water containing tetramethylsilane (TMS) as a reference was added to the samples using a coaxial insert.

Table S11. Europium concentrations in the aqueous phase (with 0.1 mol·dm^{-3} HNO_3 and 3.9 mol·dm^{-3} $NaNO_3$ at varying Eu^{3+} concentrations) before and after contact with the TOPO–decanoic acid hydrophobic eutectic solvent, together with the corresponding europium concentrations in the HES phase

**Eu^{3+} concentration in aqueous phase before extraction**						
**$[Eu(NO_3)_3{\cdot}nH_2O]$ / g·dm^{-3}**	1	5	10	20	30	50
**$[Eu]_{ini,aq}$ / g·dm^{-3}**	0.237	1.76	2.22	6.96	8.92	18.2
**$[Eu]_{ini,aq}$ x 10^2 / mol·dm^{-3}**	0.156	1.16	1.46	4.58	5.87	11.99
**Aqueous and HES phase Eu concentrations after extraction (FTIR samples)**						
**$[Eu]_{f,aq}$ x 10^3 / g·dm^{-3}**	0.183	0.538	1.36	11.2	62.4	727
**$[Eu]_{f,aq}$ x 10^5 / mol·dm^{-3}**	0.120	0.354	0.897	7.37	41.1	479
**$[Eu]_{f,HES}$ / g·dm^{-3}**	0.473	3.51	4.44	13.9	17.7	34.9
**$[Eu]_{f,HES}$ x 10^2 / mol·dm^{-3}**	0.311	2.31	2.92	9.15	11.7	23.0
**Aqueous and HES phase Eu concentrations after extraction (NMR samples)**						
**$[Eu]_{f,aq}$ x 10^3 / g·dm^{-3}**	-	1.14	-	-	-	809
**$[Eu]_{f,aq}$ x 10^5 / mol·dm^{-3}**	-	0.750	-	-	-	533
**$[Eu]_{f,HES}$ / g·dm^{-3}**	-	3.51	-	-	-	34.8
**$[Eu]_{f,HES}$ x 10^2 / mol·dm^{-3}**	-	2.31	-	-	-	22.9

FTIR

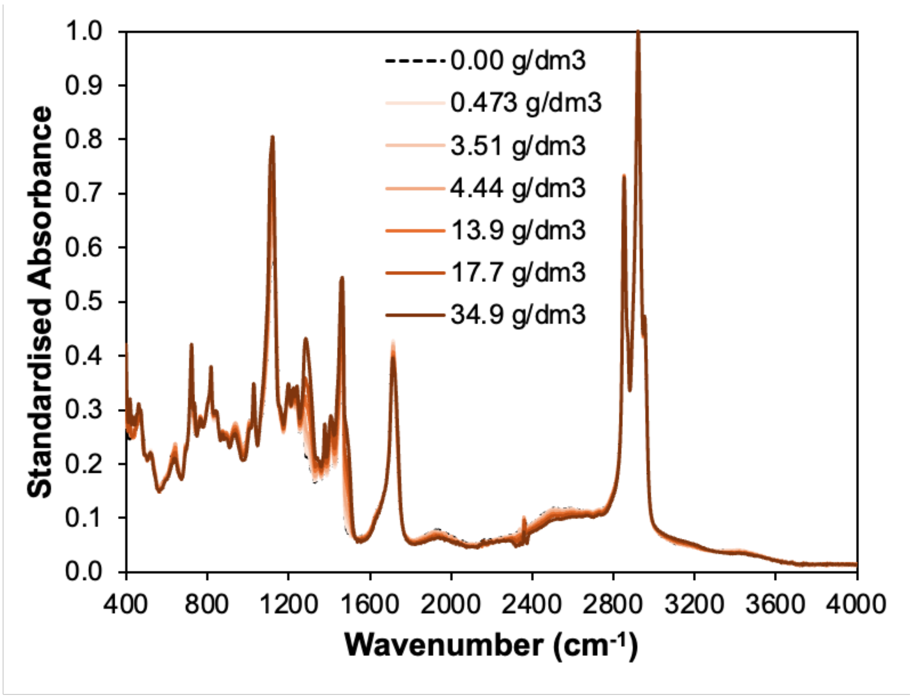

Figure S9. FTIR spectra of the TOPO+Decanoic acid HES phase for x_{TOPO}=0.5 after contact with an aqueous phase containing 4.0 mol·dm^{-3} nitrate anion (0.1 mol·dm^{-3} HNO_3 with 3.9 mol·dm^{-3} $NaNO_3$) as a function of the equilibrium HES phase concentration $[Eu^{3+}]_{HES}$ from 0 to 35 g·dm^{-3}.

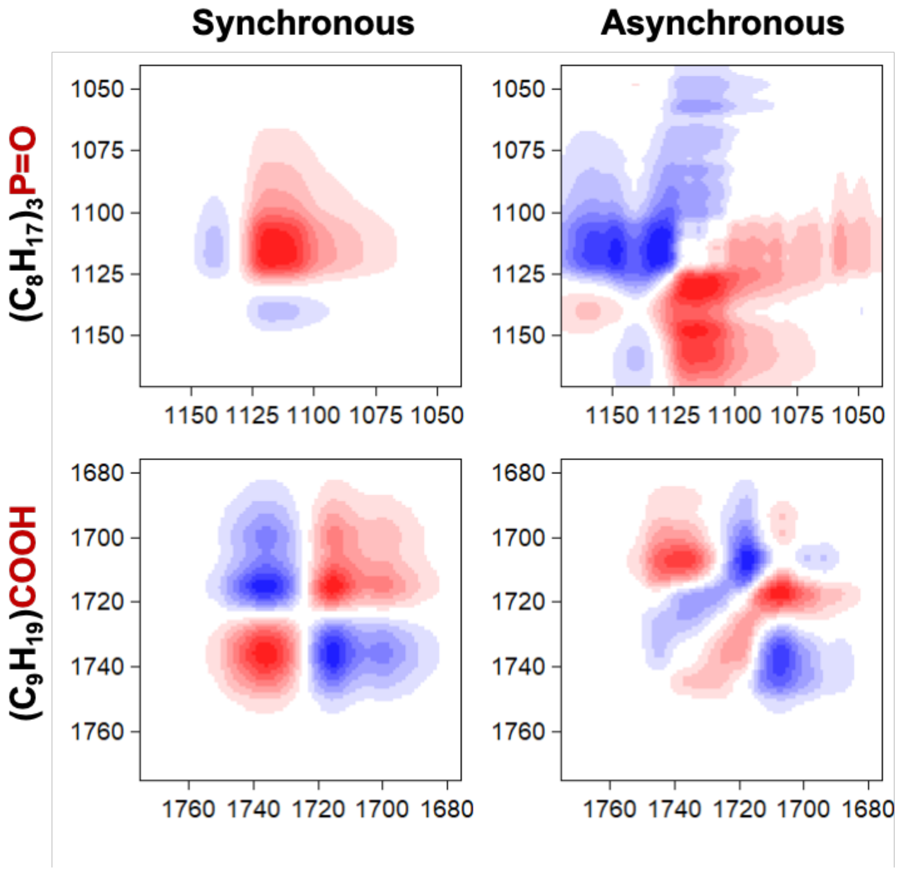

Figure S10. Synchronous and asynchronous FTIR two-dimensional correlation spectroscopy analysis of the HES bands shown in **Figure 3** of the manuscript as a function of the equilibrium HES phase concentration $[Eu^{3+}]_{HES}$ from 0 to 35 g·dm^{-3}.

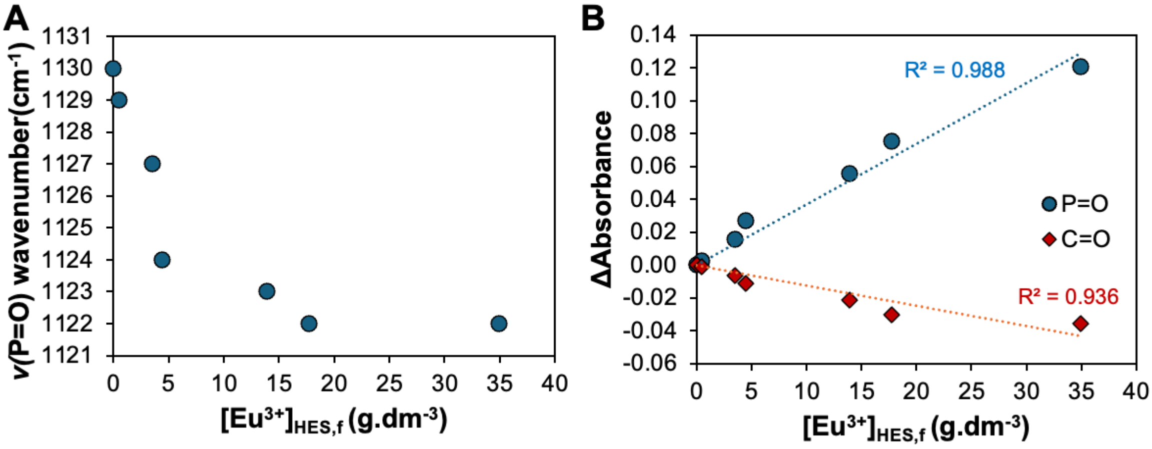

Figure S11. A) Shift in the P=O stretch vibration of TOPO and B) change in the absorbance of the P=O and C=O stretch vibrations of TOPO and decanoic acid respectively in the TOPO+decanoic acid HES phase for x_{TOPO}= 0.5 as a function of the equilibrium HES phase concentration $[Eu^{3+}]_{HES}$ from 0 to 35 g·dm^{-3}.

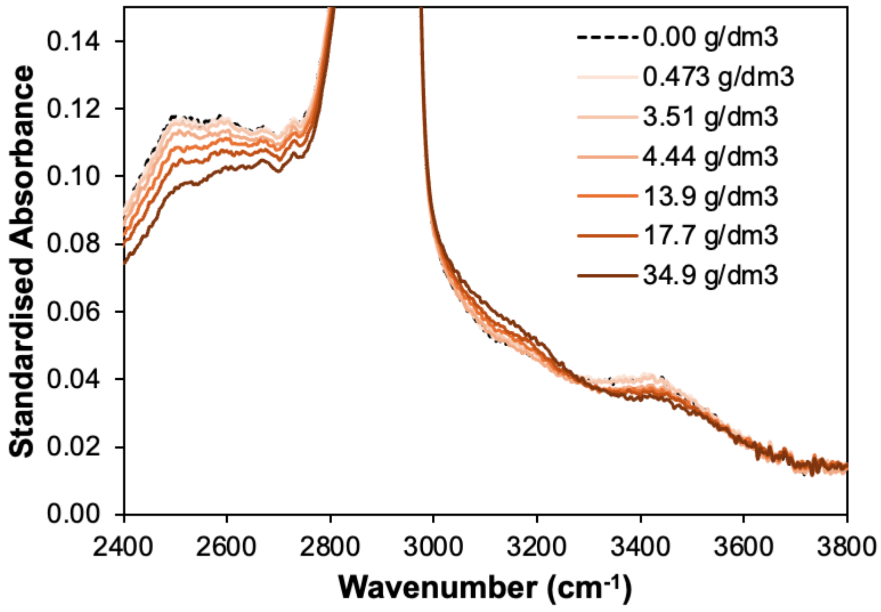

Figure S12. FTIR spectra of the TOPO+Decanoic acid HES focusing on the OH stretch region. Conditions are the same as described in **Figure S10.**

NMR

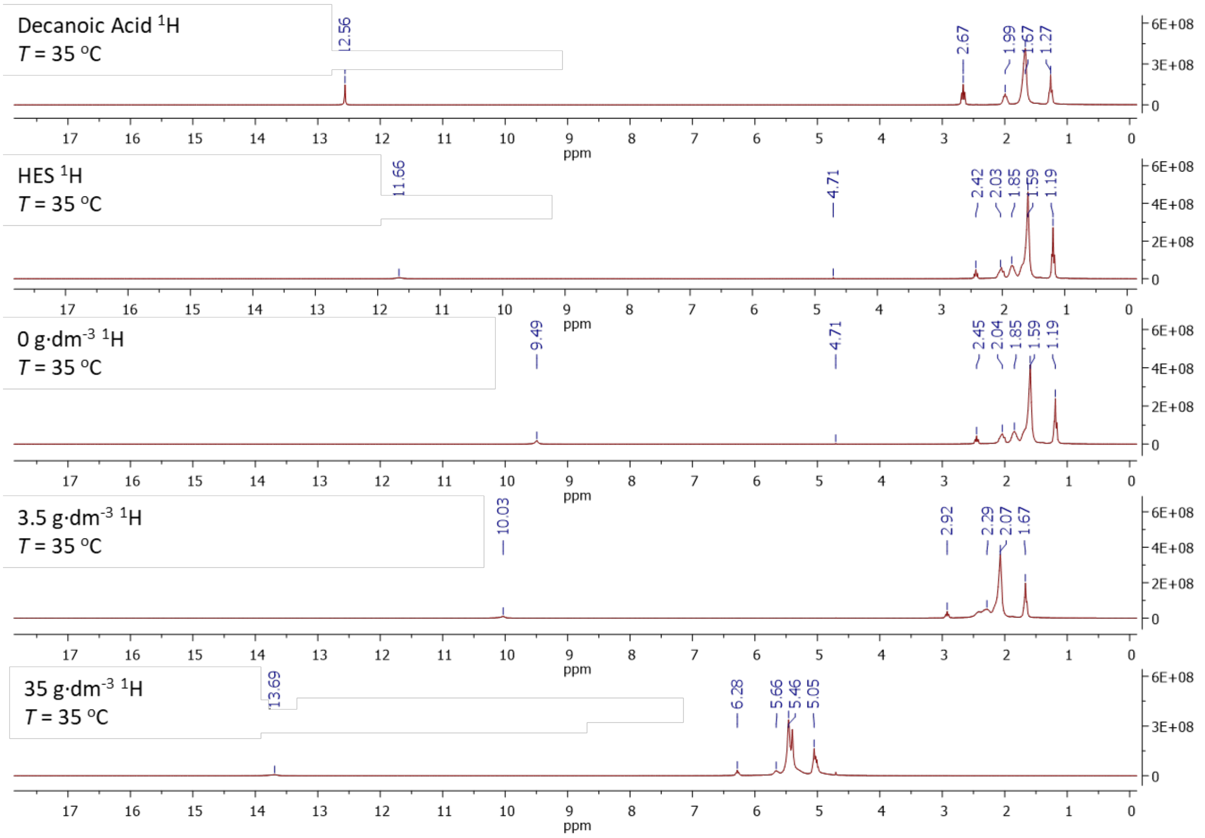

Figure S13. ^{1}H NMR spectra of decanoic acid, TOPO+Decanoic acid HES as-prepared, HES after aqueous phase equilibration ($[Eu^{3+}]_{HES}$ = 0 g·dm^{-3}), and after europium extraction at two levels of concentration of europium in the aqueous phase to yield $[Eu^{3+}]_{HES}$ = 3.5 g·dm^{-3} and $[Eu^{3+}]_{HES}$ =35 g·dm^{-3}.

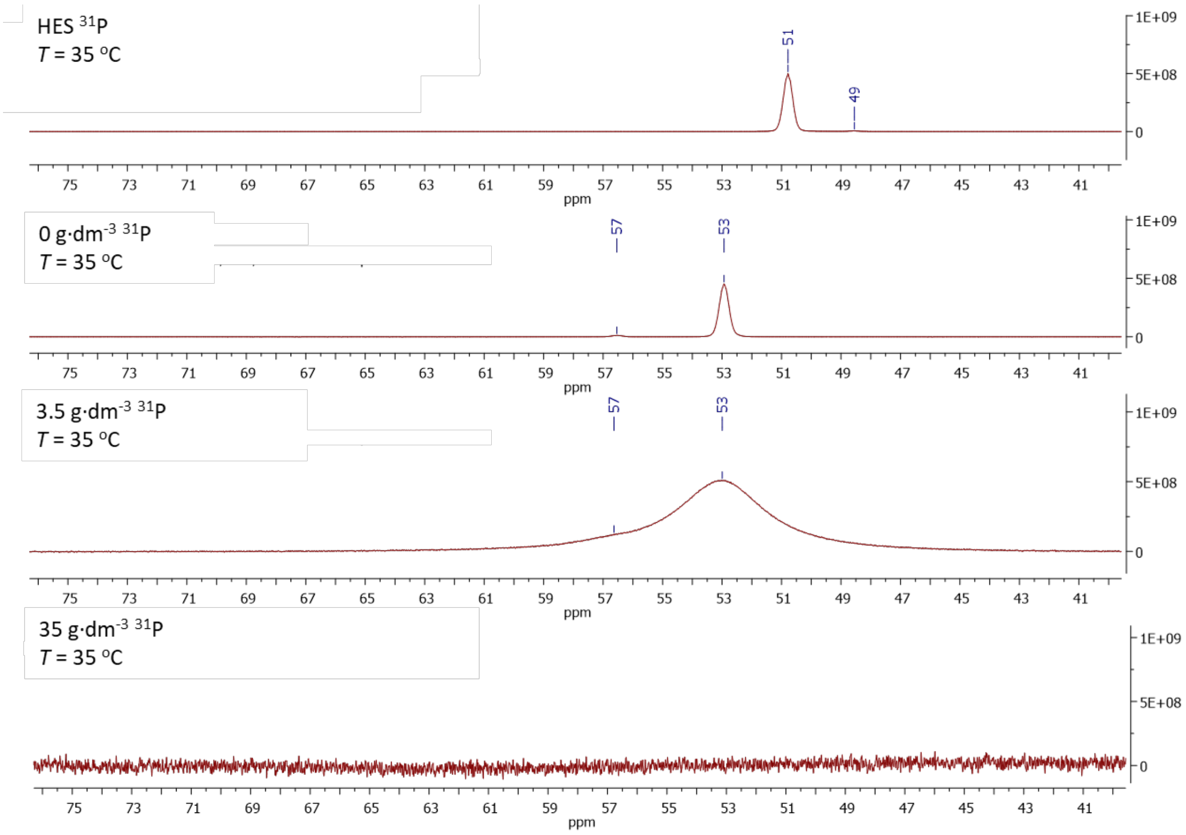

Figure S14. ^{31}P NMR spectra of the TOPO+Decanoic acid HES as-prepared, HES after aqueous phase equilibration ($[Eu^{3+}]_{HES}$ = 0 g·dm^{-3}), and after europium extraction at two levels of concentration of europium in the aqueous phase to yield $[Eu^{3+}]_{HES}$ = 3.5 g·dm^{-3} and $[Eu^{3+}]_{HES}$ =35 g·dm^{-3}.

References

1 N. Schaeffer, J. H. F. Conceiçaõ, M. A. R. Martins, M. C. Neves, G. Pérez-Sánchez, J. R. B. Gomes, N. Papaiconomou and J. A. P. Coutinho, *Green Chem*, 2020, **22**, 2810–2820.

2 T. S. Grimes, P. R. Zalupski and L. R. Martin, *J Phys Chem B*, 2014, **118**, 12725–12733.

3 L. M. N. B. F. Santos, M. T. Silva, B. Schröder and L. Gomes, *J Therm Anal Calorim*, 2007, **89**, 175–180.

4 I. C. M. Vaz, A. Bhattacharjee, M. A. A. Rocha, J. A. P. Coutinho, M. Bastos and L. M. N. B. F. Santos, *Phys Chem Chem Phys*, 2016, **18**, 19267–19275.

5 I. Wadsö and R. N. Goldberg, *Pure Appl Chem*, 2001, **73**, 1625–1639.

6 M. I. Davis and E. S. Ham, *Thermochim. Acta*, 1991, **190**, 251–258.

7 S. H. Tanaka, H. I. Yoshihara, A. W. C. Ho, F. W. Lau, P. Westh and Y. Koga, *Can J Chem*, 1996, **74**, 713–721.

8 D. M. Petkovlć, M. M. Kopečni and & A. A. Mltrovlć, *Solvent Extr Ion Exch*, 1992, **10**, 685–696.

9 M. Alibrahim and H. Shlewit, *Liquid-liquid extraction of nitric acid by TOPO/dodecane*, Syrian Arab Republic, 2007. Available at: https://inis.iaea.org/records/ar0x1-fpy33

10 R. A. Zingaro and J. C. White, *J Inorg Nucl Chem*, 1960, **12**, 315–326.

11 G. Suresh, M. S. Murali and J. N. Mathur, *Radiochim. Acta*, 2003, **91**, 127–134.

12 W. J. Hamer and Y. Yung chi, *J. Phys. Chem. Ref. Data*, 1972, **1**, 1047–1100.

13 A. Klamt, *J Phys Chem*, 1995, **99**, 2224–2235.

14 A. Klamt and F. Eckert, *Fluid Phase Equilib.*, 2000, **172**, 43–72.

15 Dassault Systèmes. BIOVIA COSMOtherm. 2021. http://www.3ds.com

16 TURBOMOLE V7.4. *A Development of University of Karlsruhe and Forschungszentrum Karlsruhe GmbH, 1989–2007, TURBOMOLE GmbH*, 2019, available from https://www.turbomole.org/.

17 J. P. Perdew, *Phys. Rev. B*, 1986, **33**, 8822–8824.

18 E. Caldeweyher, J. M. Mewes, S. Ehlert and S. Grimme, *Phys Chem Chem Phys*, 2020, **22**, 8499–8512.

19 F. Weigend and R. Ahlrichs, *Phys Chem Chem Phys*, 2005, **7**, 3297–3305.

20 A. Hellweg and F. Eckert, *AIChE Journal*, 2017, **63**, 3944–3954.

21 M. Newville, *J. Phys. Conf. Ser.*, 2013, **430**, 012007.

22 A. L. Ankudinov, B. Ravel, J. J. Rehr and S. D. Conradson, *Phys. Rev. B*, 1998, **58**, 7565.

23 A. Bowden, K. Singh and A. W. G. Platt, *Polyhedron*, 2012, **42**, 30–35.
